# Selection and Trans-Species Polymorphism of Major Histocompatibility Complex Class II Genes in the Order Crocodylia

**DOI:** 10.1371/journal.pone.0087534

**Published:** 2014-02-04

**Authors:** Weerachai Jaratlerdsiri, Sally R. Isberg, Damien P. Higgins, Lee G. Miles, Jaime Gongora

**Affiliations:** 1 Faculty of Veterinary Science, University of Sydney, Sydney, New South Wales, Australia; 2 Centre for Crocodile Research, Noonamah, Northern Territory, Australia; 3 Faculty of Veterinary Science, University of Sydney, Sydney, New South Wales, Australia; CSIRO, Australia

## Abstract

Major Histocompatibility Complex (MHC) class II genes encode for molecules that aid in the presentation of antigens to helper T cells. MHC characterisation within and between major vertebrate taxa has shed light on the evolutionary mechanisms shaping the diversity within this genomic region, though little characterisation has been performed within the Order Crocodylia. Here we investigate the extent and effect of selective pressures and trans-species polymorphism on MHC class II *α* and *β* evolution among 20 extant species of Crocodylia. Selection detection analyses showed that diversifying selection influenced MHC class II *β* diversity, whilst diversity within MHC class II *α* is the result of strong purifying selection. Comparison of translated sequences between species revealed the presence of twelve trans-species polymorphisms, some of which appear to be specific to the genera *Crocodylus* and *Caiman*. Phylogenetic reconstruction clustered MHC class II *α* sequences into two major clades representing the families Crocodilidae and Alligatoridae. However, no further subdivision within these clades was evident and, based on the observation that most MHC class II *α* sequences shared the same trans-species polymorphisms, it is possible that they correspond to the same gene lineage across species. In contrast, phylogenetic analyses of MHC class II *β* sequences showed a mixture of subclades containing sequences from Crocodilidae and/or Alligatoridae, illustrating orthologous relationships among those genes. Interestingly, two of the subclades containing sequences from both Crocodilidae and Alligatoridae shared specific trans-species polymorphisms, suggesting that they may belong to ancient lineages pre-dating the divergence of these two families from the common ancestor 85–90 million years ago. The results presented herein provide an immunogenetic resource that may be used to further assess MHC diversity and functionality in Crocodylia.

## Introduction

The Major Histocompatibility Complex (MHC) contains highly variable multi-gene families, which play a key role in the adaptive immune response within vertebrates. MHC characterisation and comparison across major vertebrate taxa have provided valuable insights into vertebrate evolution since the MHC evolved from primitive species, such as sharks, approximately 472–584 million years ago (MYA) [1]–[4]. Comparison of closely related species has revealed that changes in MHC gene content, number and organisation often occur in antigen presentation genes and this is relevant for an investigation of rapid MHC diversification, and gene loss and gain in a short evolutionary timeframe [5]–[7]. However, such comparative studies in non-avian reptiles, specifically the Order Crocodylia, are limited. Here we investigate to what extent diversification processes have played a role in the evolution of MHC class II *α* and *β* nucleotide sequences across 20 extant species of Crocodylia.

MHC genes are grouped into three classes. One of these, class II, is involved primarily in the presentation of extracellular peptides to helper T-cells [8]. The molecules encoded by MHC class II genes consist of *α* and *β* chains, each of which is organised into two extracellular domains (*α*1/*α*2 and *β*1/*β*2 respectively), one transmembrane and one cytoplasmic tail domain [9]. Typically, MHC class II *α* and II *β* genes are organised into four to six exons: exon 1 encoding the leader sequence, exons 2 and 3 encoding the two extracellular domains, and exon 4 encoding the transmembrane domain with some variable exons (exons 5 and 6) encoding for the cytoplasmic tail and the 3′-untranslated region ([10], [11], reviewed in [12]).

MHC class II genes are polymorphic and polygenic, thus generating diversity for loading and presentation of antigens from a wide range of microorganisms [13]. The high degree of polymorphism of these genes is attributed partially to diversifying selection, whereby genotypes conferring a fitness advantage on the organism are maintained [14]. This could lead to more MHC variation within [7], [15] and across species [16], [17] to cope with different antigenic challenges. This selection is reflected in the predominance of nonsynonymous (dN) over synonymous (dS) substitution rates; where the ratio (*ω* = dN/dS) is expected to be more than one. Some of the polymorphism persists longer than expected among species under neutrality because *i*) new species generally inherit an appreciable endowment of MHC variants from their ancestors, *ii*) some particular sequence polymorphisms at nucleotide or amino acid level can persist after speciation and sequence divergence, and *iii*) new MHC variants are slow to accumulate [18]–[20]. The retention of nucleotide and amino acid variants across related species has been defined as ‘trans-species polymorphism (TSP)’ [20]. However, if there is low nonsynonymous, relative to synonymous, polymorphism between MHC variants amongst species, whereby functional constraints in antigen loading and presentation make MHC sequences invariable with *ω* close to zero, it is considered a signature of strong purifying selection [17].

In non-avian reptiles (lepidosaurs, testudines, and crocodilians), studies on MHC class II genes are limited to a few taxa. One study of MHC class II *β* exon 3 (*DAB1-DAB5* genes) in Galapagos marine iguanas [21] identified a greater number of gene loci than in other vertebrates suggesting independent duplication events from a small number of ancestral genes [21]. The only species of Crocodylia for which studies related to the MHC class II have been undertaken thus far are the Chinese alligator (*Alligator sinensis*) and Nile crocodile (*Crocodylus niloticus*), both of which showed some diversity of MHC genes at the population level [22], [23]. Additional MHC class II studies across species of Crocodylia are required to understand the evolutionary mechanisms that have driven and maintained diversity within this class of genes. Although there is an ongoing initiative to sequence representative genomes from each of three extant families of Crocodylia (Alligatoridae, Crocodilidae and Gavialidae) [24], further data on the MHC for other species of Crocodylia still needs to be generated for comparative analyses. To address this gap, we investigated to what extent diversifying selection and trans-species polymorpism have played a role in the evolution of MHC class II *α* and *β* genes across 20 species of Crocodylia. Overall, we identified ancient trans-species polymorphisms within and between families of the Order Crocodylia, and detected a greater role of diversifying selection in the MHC class II *β* relative to the MHC class II *α*.

## Results

### Characterisation of MHC Class II *α* and *β*

Eighteen and eleven sequences of MHC class II *α* exons 2 and 3, respectively, were identified among species of the Order Crocodylia (Table 1; Figure S1). Single sequences per specimen and species were obtained for both exons 2 and 3. Whilst these data might suggest that only a single locus is present for each species, the presence of additional undetected gene copies within these species cannot be excluded. Seventy-two sequences (260 bp) of MHC class II *β* exon 3 were identified among 20 species of Crocodylia (Table 1; Figure S2). Between one and four sequences per individual within a species were identified, suggesting that at least two loci were being amplified in the current study. More details regarding characterisation of MHC class II *α* and *β* are described in Appendix S1 and Appendix S2.

**Table 1:** Summary of 20 species of Crocodylia investigated in the current study for MHC class II *α* exons 2 and 3 plus MHC class II *β* exon 3, their assigned gene prefixes, and numbers of sequences per species or per individual.

	Gene	Class II *β* exon 3		Class II *α* exon 2		Class II *α* exon 3		
Species^a^	prefix	NS (NC)^b^	SPI (N)^c^	NS (NC)	SPI (N)	NS (NC)	SPI (N)	GenBank accession numbers
**Crocodilidae**								
Freshwater crocodile	*Crjo*	4 (9)	1 and 3	1 (4)	1 (1)	NA^d^	NA	GU126804-07, GU126954
(*Crocodylus johnsoni*)			(2)					
Philippine crocodile	*Crmi*	2 (4)	2 (1)	1 (4)	1 (1)	1 (4)	1 (1)	GU126810-11, GU126950,
(*Crocodylus mindorensis*)								GU126959
Nile crocodile	*Crni*	3 (8)	1 and 2	1 (4)	1 (1)	NA	NA	GU126822-23, GU126825, GU126929
(*Crocodylus niloticus*)			(2)					
American crocodile	*Crac*	5 (9)	2 and 3	1 (4)	1 (1)	1 (4)	1 (1)	GU126827-30, GU126832,
(*Crocodylus acutus*)			(2)					GU126934, GU126960
Mugger crocodile	*Crpa*	2 (4)	2 (1)	1 (4)	1 (1)	1 (4)	1 (1)	GU126833, GU126835, GU126942,
(*Crocodylus palustris*)								GU126958
Dwarf crocodile	*Oste*	5 (9)	1 and 4	1 (4)	1 (1)	1 (4)	1 (1)	GU126836, GU126839-41,
(*Osteolaemus tetraspis*)			(2)					GU126843, GU126936, GU126961
Siamese crocodile	*Crsi*	3 (8)	1 and 3	1 (4)	1 (1)	NA	NA	GU126944, GU126846-48
(*Crocodylus siamensis*)			(2)					
Slender-snouted crocodile	*Meca*	4 (10)	1 and 4	1 (4)	1 (1)	NA	NA	GU126849, GU126851, GU126855-
(*Mecistops cataphractus*)			(2)					56, GU126931
Orinoco crocodile	*Crin*	4 (8)	2 (2)	NA	NA	1 (4)	1 (1)	GU126890-91, GU126893,
(*Crocodylus intermedius*)								GU126895, GU126957
Cuban crocodile	*Crrh*	4 (8)	1 and 3	1 (4)	1 (1)	NA	NA	GU126896-97, GU126899,
(*Crocodylus rhombifer*)			(2)					GU126900, GU126951
New Guinea crocodile	*Crno*	2 (4)	2 (1)	1 (4)	1 (1)	NA	NA	GU126909, GU126911, GU126953
(*Crocodylus novaeguineae*)								
Saltwater crocodile	*Crpo*	4 (10)	1 and 3	1 (4)	1 (1)	1 (4)	1 (1)	GU126912-13, GU126915,
(*Crocodylus porosus*)			(2)					GU126919, GU126967
Morelet's crocodile	*Crmo*	5 (9)	2 and 3	1 (4)	1 (1)	1 (4)	1 (1)	GU126920-21, GU126923,
(*Crocodylus moreletii*)			(2)					GU126927-28, GU126938, GU126962
**Alligatoridae**								
American alligator	*Almi*	4 (10)	1 and 3	1 (4)	1 (1)	NA	NA	GU126813, GU126815-17, GU126940
(*Alligator mississippiensis*)			(2)					
Chinese alligator	*Alsi*	4 (10)	2 and 3	NA	NA	1 (4)	1 (1)	GU126880-83, GU126963
(*Alligator sinensis*)			(2)					
Cuvier's dwarf caiman	*Papa*	3 (8)	0^e^and 3	1 (4)	1 (1)	NA	NA	GU126858-59, GU126861, GU126941
(*Paleosuchus palpebrosus*)			(2)					
Spectacled caiman	*Cacr*	2 (8)	0 and 2	1 (4)	1 (1)	1 (4)	1 (1)	GU126865, GU126867, GU126945,
(*Caiman crocodylus*)			(2)					GU126966
Broad-snouted caiman	*Cala*	4 (9)	1 and 3	1 (4)	1 (1)	1 (4)	1 (1)	GU126871, GU126873, GU126877-
(*Caiman latirostris*)			(2)					78, GU126948, GU126964
Yacare caiman	*Caya*	5 (8)	2 and 3	1 (4)	1 (1)	NA	NA	GU126903-05, GU126907-08,
(*Caiman yacare*)			(2)					GU126955
Black caiman	*Meni*	3 (8)	1 and 3	1 (4)	1 (1)	1 (4)	1 (1)	GU126887-89, GU126939, GU126965
(*Melanosuchus niger*)			(2)					

a Common names and their scientific names for the Order Crocodylia in brackets.

b NC in brackets indicates the number of clone inserts sequenced from which number of sequences within each species (NS) were identified. The NS values also include pseudogenes, and are discussed in the results section.

c SPI indicates number of sequence(s) per individual with the number of individuals (N) examined in brackets. The SPI and N values are discussed in the results section.

d Not applicable due to unsuccessful results from a process of cloning.

e Zero means none of the sequences observed in an individual after eliminating sequence artefacts described below.

### Trans-species Polymorphism and Non-functional Sequences

Four, seven and forty-two translated sequences were identified for MHC class II *α* exons 2 and 3 and II *β* exon 3, respectively. Based on identical amino acid sequences between species, twelve trans-species polymorphisms (named TSP1 through TSP12) were identified among all the amino acid sequences: two in each of the MHC class II *α* exons and eight in the MHC class II *β* exon 3 (Table 2; Figures S1 and S2). TSP1 from MHC class II *α* exon 2 was detected in 13 species from six genera of Crocodylia, while TSP3 and TSP4 from MHC class II *α* exon 3 were observed only among species from the genus *Crocodylus* (Table 2). TSP5 from MHC class II *β* exon 3 was identified in 11 of the 20 species studied. Finally, TSP6-12 were observed in two to six species each.

**Table 2:** Number of species possessing a particular TSP across families and genera of Crocodylia.

		Family							
		Crocodilidae			Alligatoridae				
		Genus^c^							
TSP^a^	N^b^	Cro	Mec	Ost	All	Cai	Pal	Mel	MHC sequence^d^
II *α* exon 2									
TSP1	13	7	1	0	1	2	1	1	*Crmi-DA01, Crni-DA01, Crac-DA01, Crpa-DA01, Crsi-DA01, Crno-DA02, Crpo-DA01, Meca-DA01, Almi-DA01, Papa-DA01, Cacr-DA01, Cala-DA01, Meni-DA01*
TSP2	3	2	0	0	0	1	0	0	*Crrh-DA01, Crmo-DA02, Caya-DA01*
II *α* exon 3									
TSP3	2	1	0	1	0	0	0	0	*Oste-DA03, Crin-DA01*
TSP4	4	4	0	0	0	0	0	0	*Crmi-DA02, Crac-DA02, Crpa-DA02, Crpo-DA01*
II *β* exon 3									
TSP5	11	8	1	1	1	0	0	0	*Crjo-DB04, Crac-DB04, Crpa-DB03, Crsi-DB04, Crin-DB04&06, Crrh-DB04, Crpo-DB04, Crmo-DB04, 08&09, Oste-DB04&08, Meca-DB07, Alsi-DB04*
TSP6	6	1	1	0	2	1	1	0	*Crni-DB03, Meca-DB03&08, Papa-DB03, Cala-DB03, Alsi-DB03, Almi-DB03*
TSP7	4	3	0	1	0	0	0	0	*Oste-DB01, Crac-DB01, Crpa-DB01, Crin-DB01*
TSP8	3	0	0	0	0	2	1	0	*Papa-DB02, Cacr-DB02, Caya-DB02*
TSP9	3	2	0	0	0	0	0	1	*Crin-DB02, Crrh-DB02, Meni-DB02*
TSP10	2	0	0	0	0	2	0	0	*Cala-DB01, Caya-DB01*
TSP11	2	2	0	0	0	0	0	0	*Crjo-DB01, Crpo-DB01*
TSP12	2	2	0	0	0	0	0	0	*Crjo-DB02, Crac-DB02*

a TSPs that are translated from nucleotide sequences (last column), and that are observed among species with the same or different genera of Crocodylia.

b Total number of species possessing the correspondent TSP.

c Number of species within the following genera that share the same TSP: *Crocodylus* (Cro); *Mecistops* (Mec); *Osteolaemus* (Ost); *Alligator* (All); *Caiman* (Cai); *Paleosuchus* (Pal); *Melanosuchus* (Mel).

d Nucleotide sequences that are deduced into the same TSP.

In total, 11 non-functional sequences were found among the MHC class II exons studied (Figures S1 and S2). Interestingly, a single base pair (bp) deletion at amino acid (aa) site 12, resulting in a break in the open reading fragment, appears to be shared by three MHC class II *α* exon 2 sequences (*Crrh-DA01*, *Crmo-DA02* and *Caya-DA01*) from *C. rhombifer*, *C. moreletii* and *C. yacare*. In addition, stop codons at aa site 31 or 48 were observed among three MHC class II *α* exon 3 sequences (*Crin-DA01*, *Oste-DA03* and *Cacr-DA02*) from *C. intermedius*, *O. tetraspis* and *C. crocodylus* (Figure S1). Despite the same individual being investigated for each species, it is difficult to conclude that the exons identified belong to a single gene due to the possibility of polygeny [8]. Apparently, the species in which the non-functional sequences of MHC class II *α* were identified did not have additional sequences with intact open reading fragments; it appears likely that those sequences may have been missed during the screening of clones as most species were found to possess intact MHC class II *α* coding sequences. For the MHC class II *β* exon 3, five non-functional sequences from four species of Crocodilidae (*Crrh-DB02*, *Crno-DB01*, *Crmo-DB01* and *Crin-*DB02) and a single species of Alligatoridae (*Meni-DB02*) were identified, based on the presence of stop codons. However, other sequences from these five species displayed functional putative reading fragments with most containing conserved peptide interacting sites and cysteine bridges (C-C).

### Effect of Diversifying Selection on MHC Class II *α* and *β*

**Figure 1:**
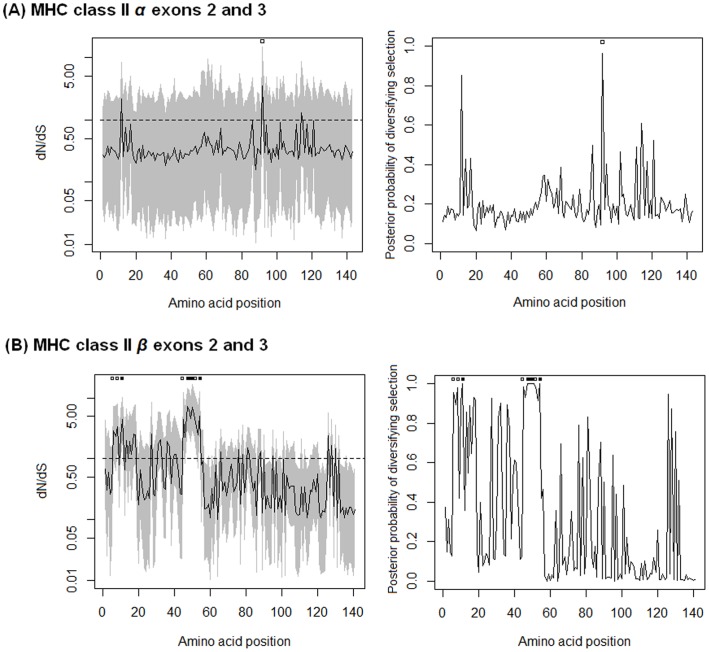
Selection detection analyses of MHC class II *α* and *β* across species of Crocodylia. Tests were performed by Bayesian inference of (A) MHC class II *α* exons 2 and 3 sequences, and (B) MHC class II *β* exons 2 and 3 sequences. Graphs show spatial change in dN/dS ratios (*ω*) and the posterior probabilities of diversifying selection across amino acid positions. Lines on the left-sided graphs present estimates of dN/dS ratios across amino acid positions; grey areas indicate 95% highest posterior probability dense intervals; and dashed lines show dN/dS values equal to one. Amino acid positions show significant (posterior probabilities >0.95) and highly significant diversifying selection (posterior probabilities >0.99), displayed by open squares and dark squares, respectively.

Bayesian analyses of MHC class II *β* gene sequences among species of Crocodylia showed evidence of diversifying selection on their exon 2 with high dN/dS ratios (Figure 1). Figure 1B suggests that seven amino acid sites have been selected for diversification with highly significant support (posterior probability >0.99). Of these sites (*Cacr-B1* as a reference; Figure S3), exon 2 sites 47W (dN/dS = 7.241; CI = 2.879–14.634), 50Y (7.002; 2.881–17.725), 51K (5.314; 2.369–13.262) and 54E (5.057; 2.315–13.06) corresponded to sites in the expected peptide-binding residues (PBR) of the human MHC class II *β* molecule [25], and exon 2 sites 11I (4.493; 1.995–10.564), 48M (6.361; 2.511–13.707), and 49E (4.537; 1.874–10.808) were situated within a distance of two amino acid positions of the PBR (Z-test of H_A_: dN>dS at all the PBR in Figure S3, Test statistic = 1.716; *P*-value = 0.044). The sites under diversification, as identified in our study, resemble those PBR sites in humans that are polymorphic, yielding a wide variety of motif specificities for antigen binding so as to handle the immune response to any pathogen, regardless of how peculiar the pathogenic protein might be [25].

In contrast, the Bayesian analysis of MHC class II *α* sequences did not detect sites under diversifying selection in the antigen presentation region of exon 2 (Figure 1A). Although a single exon 3 site, 92V (*Crpo-DA01* as a reference; Figure S4) had mean dN/dS equal to 3.528, with the posterior probability equal to 0.96, exon 2 did not show significant posterior probability of diversifying selection. In addition, 140 sites of exons 2 and 3 (out of 143) revealed low dN/dS values ranging from 0.160 to 0.970 (Z-test of H_A_: dN<dS, Test statistic = 2.401; *P*-value = 0.009), which is of interest for comparisons of MHC genes between species. The data analysed herein highlight a predominant role of purifying selection acting on amino acid sites of MHC class II *α*, especially peptide-binding sites, consistent with the high number of synonymous substitutions and highly conserved amino acid sites involved in forming the MHC-peptide complex described in Figure S1. Moreover, our analyses have shown that recombination does not play a major role in generating diversity among the MHC class II sequences in Crocodylia (described more in Appendix S3), thereby dispensing with any likelihood of bias caused by high recombination rates.

### Phylogenetic Reconstruction of MHC Class II *α*

**Figure 2:**
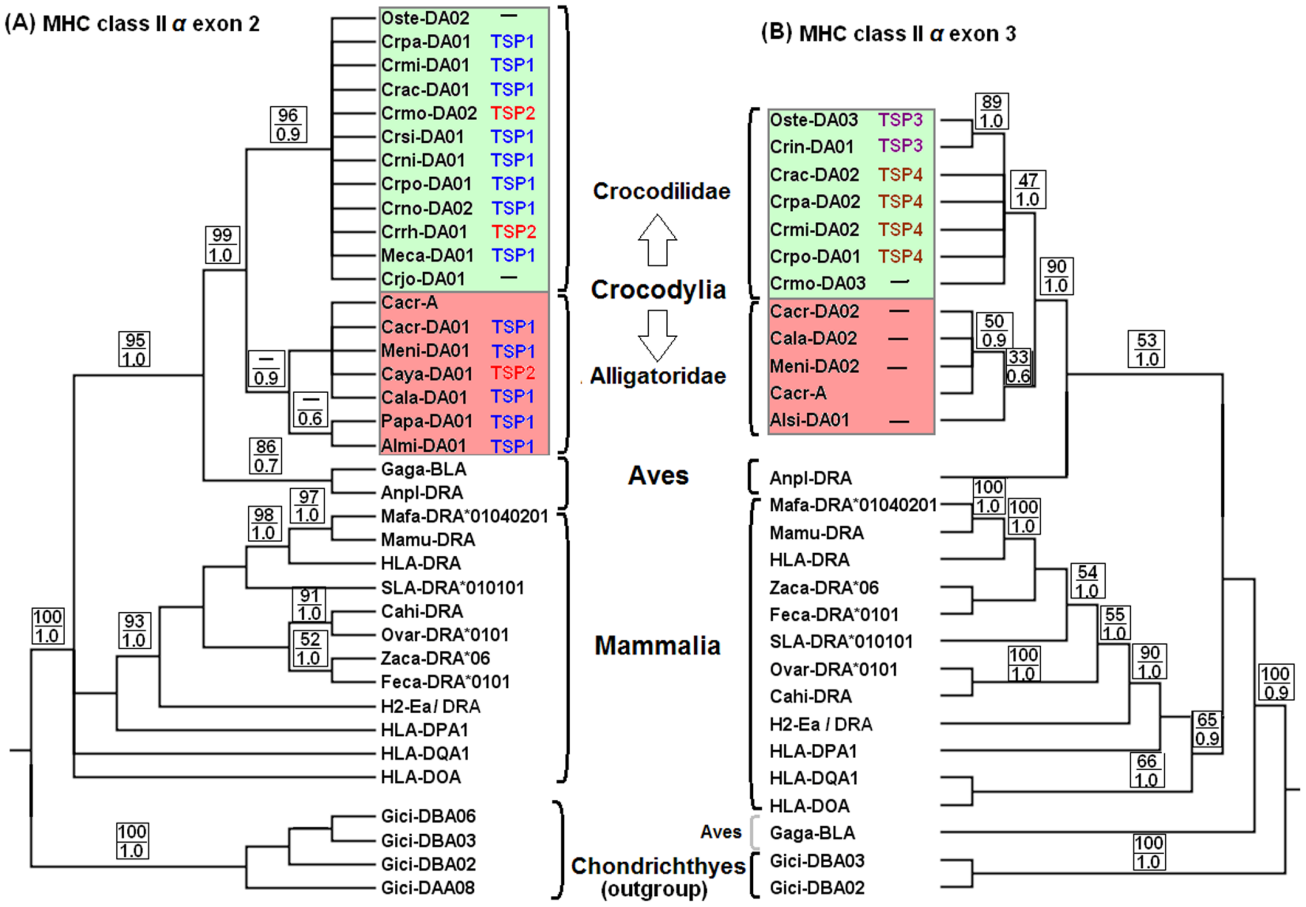
Bayesian phylogenetic trees of (A) MHC class II *α* exon 2, and (B) exon 3. Sequences of MHC class II *α* among different species of Crocodylia, Aves and Mammalia are analysed using the shark *Gici* sequences as an outgroup. Brackets in the middle show vertebrate groups to which the MHC class II *α* sequences belong. Sequences generated in the current study are from two families of Crocodylia: Crocodilidae (pale green colour) and Alligatoridae (pale red colour). TSPs associated with these sequences are described immediately after the sequence names. Support on branches is indicated by bootstrap values (BV) for maximum likelihood (above) and posterior probabilities (PP) for Bayesian analysis (below), as both analyses provide significantly similar trees described in the text. Branches with posterior probabilities below 0.5 are collapsed.

Bayesian analyses of MHC class II *α* exons 2 and 3 sequences clustered into two major clades corresponding to the families Crocodilidae and Alligatoridae (Figures 2). Maximum likelihood was consistent, with the exception of two MHC class II *α* exon 2 sequences from Alligatoridae, which clustered as sister clades of those from Crocodilidae (Figure S5-A). The expected likelihood weights (ELW) test showed that the topology of this, and the Bayesian tree, did not differ significantly (confidence tree set = 0.79 and 0.21). However, clustering of both exons within the major Crocodilidae clade did not show a clear phylogenetic signal that would permit us to determine whether subclustering was according to gene or species. Within the major Alligatoridae clade, sequences at both exons appear to cluster into two subclades representing the genera *Caiman*/*Melanosuchus* and *Alligator*/*Paleosuchus* (posterior probability (PP) = 0.9 at exon 2; 0.6 at exon 3). Furthermore, phylogenetic analyses of these MHC class II *α* sequences, as well as those from other vertebrates, showed the Crocodylia exons clustered as a sister clade of bird *DRA* (*Anpl-DRA*).

Despite clustering into two major clades, some of the amino acid TSPs, such as TSP1 and TSP2, were observed among MHC class II *α* variants in both clades. In particular, TSP1 contained an intact open reading fragment and was observed among the sequences of eight and five species of Crocodilidae and Alligatoridae, respectively (Table 2). This is likely to suggest that these sequences are orthologous, with the same functionality in the host’s immunity.

### Phylogenetic Reconstruction of MHC Class II *β*

**Figure 3:**
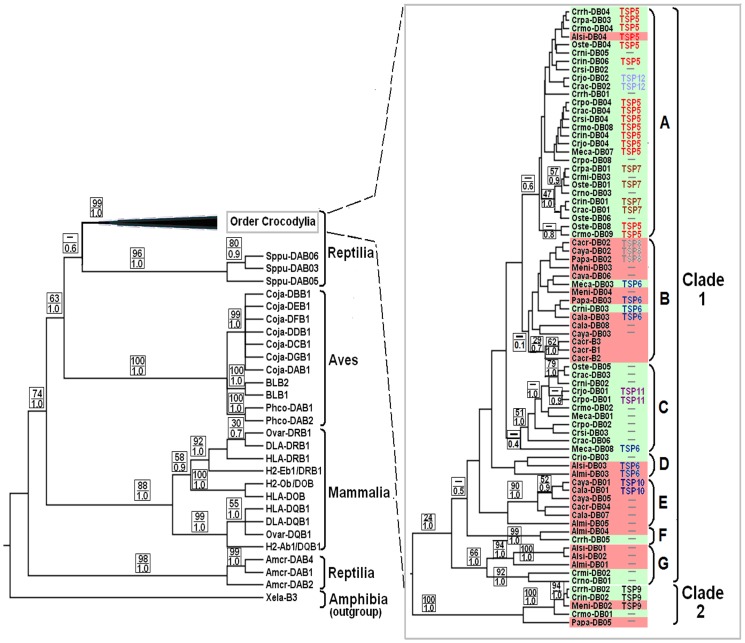
Bayesian phylogenetic trees of MHC class II *β* exon 3. This tree was constructed using sequences of MHC class II *β* among different species of Crocodylia, Aves and Mammalia using the amphibian sequence as an outgroup. Sequences generated in the current study are from two families of Crocodylia: Crocodilidae (pale green colour) and Alligatoridae (pale red colour). Brackets on the left show vertebrate groups to which the MHC class II *β* sequences belong, and those on the right show Clades 1 and 2 of the MHC sequences and seven subclades (A–G) for Clade 1. The two clades were defined on the basis of their monophyletic groupings and high posterior probability (PP = 1.0). TSP5–12 corresponding to particular MHC sequences, which are described above, follow immediately after the sequence name. Support on branches is indicated by bootstrap values (BV) for maximum likelihood (above) and posterior probabilities (PP) for Bayesian analysis (below), as both analyses provide significantly similar trees described in the text.

Bayesian and maximum-likelihood analyses of MHC class II *β* sequences from Crocodylia showed two clades (Clades 1 and 2), using tuatara sequences as outgroups (Figures 3 and S6). These two clades were differentiated by ten diagnostic sites (Figure S7). The Bayesian tree showed seven subclades in Clade 1 (1A–1G). The number of these subclades was greater than, and topology different to, those detected by the maximum-likelihood analysis, but the ELW test showed that these two trees did not differ significantly (confidence tree set = 0.67 and 0.33). Clades and subclades consisted of sequences from either/both Crocodilidae and/or Alligatoridae. Some individuals had more than one MHC class II *β* locus amplified (Tables S1 and S2) and this could explain why, in the Bayesian tree, some MHC sequences from the same individual split into different clades/subclades (Table S2). For example, Subclade 1A clustered together one to three sequences per individual for six Crocodilidae species, with the remaining sequences (1–2 per individual) clustered into Subclade 1C. Similarly, Subclade 1B clustered together two sequences per individual for *P. palpebrosus* and *M. niger,* while the remaining sequences (1 per individual and species) clustered into Clade 2; although one from *M. niger* is expected to be pseudogenic (containing a stop codon). The resultant topology within each clade and subclade was comprised of different species of Crocodylia, consistent with the notion that an orthologous relationship exists for a gene among related species, as described by Burri et al. [5].

Phylogenetic analyses across higher taxa showed that orthologous relationships were also present in MHC class II *β* genes among major lineages within Mammalia. For instance, *DRB1* sequences from different mammalian lineages clustered together, as did *DOB* and *DQB1* sequences (Figure 3). In contrast, when chicken, quail and pheasant sequences are compared (Figure 3), MHC class II *β* sequences appear to cluster by species rather than gene lineages in birds.

In the light of Bayesian analysis, a clear accumulation of TSP from different species of Crocodylia, into a number of subclades, occurred (Figure 3). For instance, the sequences *Alsi-DB04* and *Meni-DB02* from Alligatoridae clustered within subclades containing mainly Crocodilidae sequences. A similar situation was observed for the sequences *Crni-DB03* and *Meca-DB03* from Crocodilidae, which clustered with those from Alligatoridae. These examples support the conclusion that the TSPs identified in the present study play a role in complicating MHC class II evolution within and among families of Crocodylia.

## Discussion

### MHC Class II *α* and *β* are Subjected to Differential Selective Pressures

The current study has demonstrated that diversifying selection is driving and maintaining diversity of MHC class II *β* genes at the PBR within the Order Crocodylia. It could be suggested that a range of pathogenic challenges among species of Crocodylia (reviewed in [26]) and their demographic distributions around the world (Figure S8) have a role in this diversification process. In further support of this scenario, the identification of TSPs at MHC class II *β* shows preferential retention of some ancestral polymorphisms in Crocodylia, whereby specific allelic polymorphisms are maintained, thereby increasing diversity in the gene pool [27]. For instance, the distribution of TSP5 and TSP6 among the genera (*Crocodylus, Osteolaemus, Mecistops, Alligator, Caiman* and/*or Paleosuchus*) and between the families (Alligatoridae and Crocodilidae) indicates that MHC class II *β* polymorphism may have been present before these two families diverged from the common ancestor 85–90 million years ago [28]. However, a definitive conclusion of TSP across species of Crocodylia will require genomic sequence data from each species to avoid overestimation by assessing entire TSP exons (from the point of transcription to the polyadenylation signal). Analysis of intron data for MHC class II *β* genes will also permit confirmation of an ancient lineage of TSP exons as discussed above (plus differentiation between TSP and convergent evolution), particularly if the phylogenetic clustering of intron sequence data is consistent with that of exon sequence data in this study [29], [30].

In contrast to mechanisms seen in MHC class II *β* evolution, purifying selection appears to play a predominant role in the conservation of MHC class II *α* genes and their PBR. Low interspecific MHC variability, and TSPs of the MHC class II *α* exons, suggest that the Order Crocodylia has experienced purifying selection, whereby the slow-evolving MHC genes have been maintained by removing nonsynonymous nucleotide substitutions that are deleterious to immune protein function, consistent with that seen in birds [31] and mammals [17], [32]. In addition, the identification of only a single MHC class II *α* locus and at least two MHC class II *β* loci per species in the preliminary diversity analyses suggests that the *α* chain of the MHC molecule (II *α*) has maintained some conservation of protein structure necessary for interactions with different polymorphic *β* chains (II *β*). Primate species also show strong selection of MHC class II *α* genes (*DRA*), against any corrupt rearrangements or nonsilent mutants, in order to maintain their functional abilities to bind with all MHC class II *DRB* molecules available [33]–[36].

Based on the difference in selective pressures between MHC genes described above, MHC class II *β* genes appear to be more suitable markers for future diversity and disease-resistance association studies. They have more polymorphic sites than the MHC class II *α* (Appendices S1 and S2), especially those under diversifying selection (*ω*>1), where nonsynonymous substitutions occur at the amino acid level. As the MHC class II *β* gene encodes for an extracellular domain (*β* chain) of the MHC molecule directly involved in antigen presentation, these selected sites might have been mediated by pathogen challenges (reviewed in [37], [38]). Although false detection of selected sites among MHC sequences having a high level of recombination is possible [39], such a bias appears unlikely in the present case since the recombination rates measured in the current study are low, and the selected sites identified have shown strong statistical support.

### Further Sequence Subdivision of MHC Class II *β* Over MHC Class II *α*

MHC class II *α* and *β* in Crocodylia appear to cluster by gene rather than by species. The two major clades of MHC class II *α*, representing taxonomic families, could be interpreted as belonging to the same gene lineage, which may have emerged before Crocodilidae and Alligatoridae diverged from the common ancestor 85–90 MYA [28]. Although a level of sequence divergence was observed between these two major clades, marked levels of conservation among sequences within clades were also observed. This is unexpected for some genera of Crocodilidae (e.g. *Crocodylus* and *Osteolaemus*) and Alligatoridae (e.g. *Alligator* and *Paleosuchus*) that diverged approximately 22 and 65 MYA, respectively [28], particularly given that the MHC class II *α* exons studied herein encode for an extracellular domain of the MHC molecule (directly involved in the presentation of antigens). A possible explanation for the sequence conservation is that these MHC class II *α* exons may have been influenced by a preferential retention of similar variants among species within families, as an advantageous way to preserve a specific biological function [40]; and/or that mutation rates across the genome might be slow among species of Crocodylia. Supporting the latter, the Order Crocodylia has been shown to possess a significantly lower mutation rate than other vertebrate species, using mitochondrial DNA and the nuclear RAG-1 gene [41], [42]. However, further investigation may be warranted as a number of diversity studies, albeit using microsatellites, have shown moderate levels of mean heterozygosity (*H*_E = _0.040–0.941) between populations of *C. porosus* [43], *A. mississippiensis* [44] and *C. latirostris* [45] and high sequence divergence (nucleotide diversity = 0.152) at a population of *C. porosus* using MHC class I markers [46].

Furthermore, numerous phylogenetic subdivisions of MHC class II *β* exon 3 and their orthology of sequences from both Crocodilidae and Alligatoridae may suggest a greater number of gene lineages in this exon, relative to the MHC class II *α* exons described above. This is consistent with the varying numbers of loci that have been observed for both MHC class II *α* and *β* genes in other vertebrates [47]–[49], including birds (related taxa to Crocodylia) [10], [50]. These phylogenetic patterns could also be interpreted as gene lineages that have evolved divergently to each other, so as to represent different selective pressures and/or perform novel functions in immunity [6], [51]. All these results are in line with the evolution of MHC class II *α* discussed above. This is because the gene copy number and divergence of MHC class II *β* is a key factor for the MHC protein assembly of wide motif specificities to antigen binding and subsequent immune responses to pathogens [25]. Alternatively, it is possible that MHC class II *β* genes have different evolution when their orthologous relationships have been masked, especially in some bird lineages, due to recent gene duplication events and frequent genetic exchange between genes within a species (recombination or gene conversion) [5], [52]–[54].

Comparisons of the present results with a recent MHC class I study in the same taxa [55] suggest that the MHC in Crocodylia has undergone a differential pattern of evolution within and between gene classes. For instance, in contrast with MHC class II *α* genes, those for MHC class I seem to have been subject to independent events of duplication which have led to further gene-lineage diversity in Crocodilidae than in Alligatoridae. This is not surprising given that MHC class I molecules appear to be more diverse in structure and immune function than class II counterparts [56].

## Conclusions

From an evolutionary perspective, MHC class II *α* appears to be well conserved among species of the Order Crocodylia, while MHC class II *β* appears to have undergone a process of diversification. In this respect, diversification selection appears to have played a larger role in the evolution of MHC class II *β* relative to that of MHC class II *α*. These findings suggest marked degrees of differential evolution between the two MHC class II chains. In addition, the MHC class II *β* appears to be the most informative immunogenetic resource for future studies assessing population diversity for species of Crocodylia and for association studies between MHC and disease. Those studies will help refine estimates of variation in gene copy number and content among species and their implication in disease outbreaks in farmed and wild populations of Crocodylia.

## Materials and Methods

### Species Collection

We aimed to obtain DNA samples from all living species of Crocodylia. However, samples from only twenty extant species representing seven genera were obtained as follows: *Caiman* (3 spp.), *Melanosuchus* (1 spp.), *Paleosuchus* (1 spp.), *Alligator* (2 spp.), *Crocodylus* (11 spp.), *Mecistops* (1 spp.), *Osteolaemus* (1 spp.) (Table 1). Most of these samples were not purpose-collected for this study but rather came from materials sourced from previous studies [57], [58] so were obtained opportunistically. Some of them were originally collected under the University of Florida Institutional Animal Care and Use protocol number E423 and the University of Sydney Animal Ethics permit number N00/5–2009/3/5057.

### Primers, PCR, Cloning, and Sequencing

Two primer sets were generated (Table 3) to amplify the MHC class II *α* exons 2 and 3 (which encode *α1* and *α2* domains respectively), while published primers [22] were used to amplify MHC class II *β* exon 3 (which encodes the *β*2 domain). The former two sets were designed using the regions conserved between the spectacled caiman MHC class II *α* exons 2 and 3 (AF256650), MHC class II *α* sequences from human (*HLA-DRA*, NM_019111) and mallard (*Anpl-DRA*, AY905539) sequences available in GenBank. Similarly, MHC class II *β* exon 3 primers targeted conserved regions among caiman, chicken and frog sequences. The purpose of these primers was to amplify as many MHC class II gene loci as possible across all species of Crocodylia. MHC class II specificities of those primers were verified using BLASTN search in GenBank. The search performed against the genome drafts of *Alligator mississippiensis*, *Crocodylus porosus* and *Gangeticus gavialis* ([24]; Green et al. unpublished data) showed significant matches with putative MHC class II sequences in Crocodylia (Appendix S4), suggesting that the primers would result in an unbiased amplification of the presumed targets. Those genome resources are publicly available after this study was finalised. The exons targeted here were chosen because they correspond to the extracellular domains involved in the presentation of antigens and have been used to assess the evolution of MHC class II genes in other studies [59]–[61].

**Table 3:** Primer sets for MHC class II *α* exons 2 and 3 plus MHC class II *β* exon 3 used in the current study and their annealing temperatures in the cycling PCR.

Set	Primer^a^	Sequence (5′→ 3′)	Product	Expected product size (bp)	T_A_ (°C)^b^	Reference
1	II *α1*–F	AACGATGAGATCTTCCATGTGG	II *α* exon 2	171	67	Current study
	II *α1*–R	GATCTGGGACCGTGTGCG				
2	II *α2*–F	GTGTTTTCGGAGGACCCTGTG	II *α* exon 3	240	67	Current study
	II *α2*–R	CAGCCCCCAGTGCTCCAC				
3	M2-U	CTCAGTGAAGCCCAAGGTG	II *β* exon 3	260	61	Liu et al. (2007)
	M2-D	GGCTGCTGTGCTCCACCTGG				

a Forward (F) and reverse primers (R).

b Annealing temperature.

The three gene fragments were amplified using the same Polymerase Chain Reaction (PCR) reaction conditions in 50-µl reaction containing: 10 mM Tris–HCl, 50 mM KCl pH 8.3, 1.5 mM MgCl_2_, 160 µM each dNTP, 0.8 µM each primer, 0.5 U of 40∶1 mixture of Taq polymerase and Phusion® High-Fidelity DNA Polymerase (Finnzymes, Vantaa Finland), and 20–350 ng of genomic DNA. PCR cycling was as follows: 5 mins at 94°C, followed by 35 cycles of 94°C for 30 s, 61°C (for MHC class IIB primers) or 67°C (for MHC class II *α* primers) for 30 s, and 72°C for 40 s, with a final extension of 72°C for 10 mins. To enhance the efficiency of cloning, pre-cloning poly-A addition was performed as a final step by adding 0.25 U *Taq* polymerase to each PCR reaction and incubating at 72°C for 10 min. PCR products were gel purified using a UltraClean® GelSpin® Kit (MO BIO Laboratories, Inc., Carlsbad California), and were subsequently cloned using a TOPO TA Cloning*®* Kit (Invitrogen Australia Pty Limited, Mulgrave Victoria). Clones with positive inserts were confirmed for the expected insert size by *i*) PCR with the same conditions and primers and *ii*) cutting the inserts from the clones using the restriction enzyme *Eco*RI (TAKARA BIO INC., Otsu Shiga); and then visualisation in 1.7% agarose gels. Positive clones from the PCR or restriction enzyme digestion were randomly selected and four to six clone inserts per individual were sequenced using both plasmid M13 forward and reverse primers at the Australian Genome Research Facility (AGRF). We estimated to what extent the number of positive clones per individual sequenced here provided information on the variation of these genes at the individual level, using the simulated statistical model based on the probability *f*(*r*, *m*, *n*) for diploid genomes in the program NegativeMultinomial (http://www.lirmm.fr/~caraux/Bioinformatics/NegativeMultinomial/). This analysis showed that the number of positive clones used here accounted for up to 31.25% of total MHC variation in each individual. We considered this variation sufficient to retrieve representative variants for each species and preliminarily reconstruct the evolutionary history of these genes in Crocodylia, contrasting with MHC diversity studies where more clones with positive inserts are recommended to be analysed [62].

Two individuals per species were used for the amplification of MHC class II *β* exon 3 except for *C. mindorensis, C. palustris* and *C. novaeguineae* for which a single individual was used due to the sample availability. A single individual per species was used for MHC class II *α* exons 2 and 3 analysis because preliminary screening showed them to be highly conserved amongst the families Crocodilidae and Alligatoridae (97–100%). Also, samples from two and nine species (out of 20) characterised for the MHC class II *α* exons 2 and 3, respectively, generated non-MHC or waste sequences although a number of positive clones were sequenced. These species were marked as ‘NA’ in Table 1.

The criteria for categorising an insert sequence as a true MHC variant for downstream analyses were as follows: forward and reverse strands sequenced were consistent; the sequence was present in two or more of the clones analysed per individual; and/or the sequence was detected in more than one individual within and/or across species. To further filter out amplification and recombination artefacts potentially arising during PCR and cloning, the following criteria were applied to discard sequences: unique sequences that differed by less than 3 bp from a redundant sequence of the same PCR product as recommended by Edwards et al. [63] and Kloch et al. [64]; and sequences within an individual showing recombination signal from recombination test as described below (RDP3 Beta 34) [65]. More specifically, individuals with more than two sequences were checked for recombination and, if new sequences arose from a combination of the other sequences in the same individual, they were removed from the dataset. True sequences were then named using the gene prefixes of the species followed by DA (an abbreviation for MHC class II *α*) or DB (an abbreviation for MHC class II *β*) and then the identification number (Table 1), as recommended by Klein et al. [66].

### Datasets and Molecular Diversity Analyses

Three nucleotide sequence datasets were generated in the current study: *i*) a MHC class II *α* exon 2 dataset consisting of sequences from 18 species of Crocodylia; *ii*) a MHC class II *α* exon 3 dataset consisting of sequences from 11 species; and *iii*) a MHC class II *β* exon 3 dataset consisting of sequences from 20 species. This difference in numbers of species among datasets was the result of difficulties in retrieving those genes, as explained above. Forward and reverse nucleotide sequences were overlapped using the BioEdit Sequence Alignment Editor (Ibis Therapeutics, Carlsbad California) to generate a consensus sequence. To assess whether the retrieved sequences show identity to target loci among closely and distantly related vertebrate taxa, basic local alignment searches were performed in BLAST (http://www.ncbi.nlm.nih.gov/). Once confirmed, nucleotide sequences were translated into amino acids based on a standard genetic code in the MEGA 5.0 program [67] and published translated amino acid sequences from caiman MHC class II *α* (AF256650) and *β* (AF256651, AF256652, and AF277661). Alignments of nucleotide and deduced amino acid (aa) sequences were generated using MUSCLE 3.6 [68]. Indels (insertion-deletion) in the alignment were excluded from downstream analyses. In order to compare degrees of polymorphism within each dataset, molecular diversity indices were calculated, including a pairwise difference between sequences, and numbers of synonymous and nonsynonymous sites. The pairwise difference compares the number of nucleotide substitutions in each pair of MHC sequences using MEGA 5.0, while the number of synonymous and nonsynonymous sites was counted using DnaSP [69].

### Identification of Trans-species Polymorphisms and Non-functional Sequences

Trans-species polymorphisms (TSPs) in each MHC class II dataset were identified when identical amino acid sequences were found among two or more different species [65], [70], [71]. This TSP definition considers solely at the sequence level, not the evolutionary level where this polymorphism is a result of diversifying (balancing) selection [20]; however, selection was also examined as desribed below in Selection detection tests. Comparisons between these TSPs and phylogenetic trees based on nucleotide sequences were made in order to better visualise the sorting and/sharing of those TSPs within and among clades. Non-functional sequences were identified by the presence of stop codons and/or deletions. Putative functional MHC exons were identified when the entire exon showed a continuous open reading fragment and when conserved amino acid sites (N and C termini and disulfide bridge-forming cysteine), known to be implicated in forming the MHC-antigen binding complex, were present [56].

### Selection Detection Tests

In order to assess whether diversifying selection was present in each translated amino acid site of MHC genes studied herein, an estimate of a dN/dS ratio (*ω* value) performing Bayesian Inference was calculated on the following two alignment datasets: the MHC class II *α* alignment and MHC class II *β* alignment (Figures S3 and S4). Each dataset consisted of MHC sequences generated in the current study and those available in GenBank. Missing data, deletions, and stop codons (unknown characters) were treated as gaps to allow information from full-length sequences to be retained. Selection tests of the MHC class II *α* dataset from different species of Crocodylia were investigated using an alignment that contained 28 MHC class II *α* sequences generated herein and a caiman MHC class II *α* sequence available in GenBank (AF256650). In addition, the MHC class II *β* dataset from 20 species of Crocodylia was tested for selection using an input alignment that contained 18 GenBank exon 2 sequences (FJ886734–FJ886741 from the Nile crocodile and AY491421–AY491430 from Chinese alligator), three GenBank sequences of exons 2 and 3 (AF256651, AF256652 and AF277661 from the spectacled caiman), and 72 exon 3 sequences generated in the current study.

An analysis of Bayesian Inference was used to estimate *ω* of MHC class II *α* and *β* alignments, using omegaMap version 0.5 [72]. This method is able to infer sites under diversifying selection in the presence of recombination [15], which may be overestimated by CODEML. OmegaMap measures the selection parameter (*ω*) and the recombination rate (*ρ* = 4*N_e_*c), both of which are allowed to vary along the sequence alignment or amino acid positions. Two independent runs of omegaMap were conducted as follows: 5×10^5^ Markov chain Monte Carlo (MCMC) iterations, 5×10^4^ 0000 burn-in iterations, 10^2^ thinning iterations, codon frequency 1/61, the number of orderings equal to ten, and the *ω* model set to be independent. Results from both omegaMap runs that matched within an acceptable degree of error were subsequently interpreted, and graphics were created using R version 2.11.1 (http://www.r-project.org).

### Recombination Tests

In addition to possible technical reasons that could result in recombinant MHC sequences (artefacts) as described above, there are mechanisms of evolution that could result in natural events of recombination [73], [74]. In order to test any impact of recombination between MHC class II sequences from Crocodylia, the recombination rate across sequence length (*ρ*) and mean number of nucleotide substitutions in each alignment dataset (*θ*) were estimated and subsequently compared using omegaMap. An additional test of recombination was performed on three sequence datasets of MHC class II *α* exon 2, MHC class II *α* exon 3, and MHC class II *β* exon 3 using RDP3 Beta 34, as the test is able to identify possible recombinant sequences from different species of Crocodylia [75]. Default options were set with 10000 permutations and the cut-off of *p* = 0.05, and disentangle overlapping signals plus the Bonferroni correction for multiple comparisons were used. This approach allows recombinant sequences to be identified by comparing results from different recombination detection algorithms implemented in the program, using the following criteria: the recombinant sequence is detected by at least two algorithms; has a high consensus score of more than 60; and has a recombining portion from different parental sequences.

### Phylogenetic Inference of MHC Class II *α* and *β*

In order to assess orthologous relationships of MHC class II *α* and *β* sequences among species of Crocodylia and identify TSPs with very similar nucleotide sequences from two different species, phylogenetic analyses were performed using maximum likelihood in PHYML [76] and Bayesian method in BEAST version 1.5.4 [77]. The two methods were then compared to trace back the final relationships of all the MHC sequences using expected likelihood weights (ELW) with the 95% confidence tree set in TREE-PUZZLE program [78]. PHYML is found to generate a tree with high accuracy and speed, while BEAST allows enlargement of MCMC steps to average over tree space, and then makes a tree reliable with high posterior probability. The best fit model of molecular evolution [79] was selected using ModelGenerator version 0.85, according to both Bayesian Information Criterion (BIC) [80] and Akaike Information Criterion (AIC) [81]. Support values on branches for the maximum-likelihood and Bayesian methods were assessed with 10^4^ nonparametric bootstraps and 5×10^8^ MCMC steps (sampling every 10^4^ steps and 5×10^4^ burn-in steps) respectively.

Phylogenetic analyses described above were performed separately on the following three nucleotide sequence datasets as described in Table S3: *i*) a MHC class II *α* exon 2 dataset consisting of 19, 2 and 12 sequences from Crocodylia, Aves and Mammalia respectively as well as 4 sequences from Chondrichthyes, which was used as an outgroup; *ii*) a MHC class II *α* exon 3 dataset consisting of 12 sequences from Crocodylia, and the same sequences from Aves, Mammalia, and Chondrichthyes as the dataset described above; and *iii*) a MHC class II *β* exon 3 dataset consisting of 75 sequences from Crocodylia, 6 from other Reptilia, 11 from Aves, 10 from Mammalia, and a single sequence from Amphibia, which was used as an outgroup. The best-fitting model was selected for phylogenetic reconstruction as described above. The HKY model with a gamma distribution parameter (alpha) of 1.23 was used for the MHC class II *α* exon 2 dataset; HKY model with gamma distribution (alpha = 0.59) for the MHC class II *α* exon 3 dataset; and the TRN model with gamma distribution (alpha = 0.74) for the MHC class II *β* exon 3 dataset.

## Supporting Information

Figure S1 **Amino acid alignments of MHC class II** ***α*** **sequences within Crocodylia.** Variable positions are relative to the sequence at the top. The first column contains the names of MHC sequences from two families of Crocodylia: Crocodilidae (pale green colour) and Alligatoridae (pale red colour). The second column presents the amino acid alignment in letters. Dots represent amino acid identity to the top sequence; X letters represent unknown amino acids due to single-base deletions; asterisks represent stop codons; and numbers above the alignments represent the order of amino acid positions. Sites in boxes with closed triangles indicate conserved residues of antigen N and C termini on the peptide-binding region of the MHC class II *α* exon 2 alignment, as described in Kaufman et al. (1994); and sites in boxes linked with a line indicate the cysteine bridge (C-C) observed in the MHC class II *α* exon 3 alignment. Background colours in the alignments indicate degrees of amino acid identity: 100% in blue; 80–100% in yellow; and below 80% in white. The end of the alignments shows GenBank accession numbers. Trans-species polymorphisms (TSPs) are represented by numbers immediately after each MHC sequence. The same TSP is assigned with the same sequential number.
(PDF)

Figure S2 **Amino acid alignment of MHC class II** ***β*** **sequences across 20 species of Crocodylia.** Variable positions are relative to the sequence at the top. The first column contains the names of MHC sequences from two families of Crocodylia: Crocodilidae (pate green colour) and Alligatoridae (pale red colour). The second column presents the amino acid alignment in letters. Dots represent amino acid identity to the top sequence; X letters represent unknown amino acids due to single-base deletions; asterisks represent stop codons; and numbers above the alignments represent the order of amino acid positions. Sites 24 and 80 in boxes linked with a line indicate the cysteine bridge (C-C); and sites 42–66 in the red box indicate a CD4+ binding region. Background colours in the alignments indicate degrees of amino acid identity: 100% in blue; 80–100% in yellow; and below 80% in white. The end of the alignment shows GenBank accession numbers. Trans-species polymorphisms (TSPs) are represented by numbers immediately after each MHC sequence. The same TSP is assigned with the same sequential number.
(PDF)

Figure S3 **Amino acid alignment of MHC class II** ***β*** **exons 2 and 3 used for selection detection tests.** The first column contains the names of MHC sequences. The second column presents the amino acid alignment in letters. Question marks represent unknown amino acids, and numbers above the alignments represent the order of amino acid positions. Sites in boxes with open triangles indicate potential peptide contact residues on the peptide binding region of the HLA-DRB1 molecule based on crystallography models (Bondinas et al. 2007), and those in boxes with closed triangles indicate conserved residues of antigen N and C termini on the peptide-binding region of the MHC class II *β* molecule (Kaufman et al. 1994).
(PDF)

Figure S4 **Amino acid alignment of MHC class II** ***α*** **exons 2 and 3 used for selection detection tests.** The first column contains the names of MHC sequences. The second column presents the amino acid alignment in letters. Question marks represent unknown amino acids, and numbers above the alignments represent the order of amino acid positions. Sites in boxes with open triangles indicate potential peptide contact residues on the peptide binding region of the HLA-DRA molecule based on crystallography models (Bondinas et al. 2007), and those in boxes with closed triangles indicate conserved residues of antigen N and C termini on the peptide-binding region of the MHC class II α molecule (Kaufman et al. 1994).
(PDF)

Figure S5 **Maximum-likelihood trees of (A) MHC class II** ***α*** **exon 2, and (B) exon 3.** Sequences of MHC class II *α* among different species of Crocodylia, birds and mammals are analysed using the shark *Gici* sequences as an outgroup. Brackets in the middle show vertebrate groups to which the MHC class II *α* sequences belong. Bootstrap values (BV) over 50 are provided on branches. ELW tests show that these two trees did not differ significantly from their Bayesian trees of MHC class II *α* exon 2 sequences described in Fig. 2A (confidence tree set = 0.79 and 0.21), and MHC class II *α* exon 3 sequences described in Fig. 2B (confidence tree set = 0.62 and 0.38).
(PDF)

Figure S6 **Maximum-likelihood analysis of MHC class II** ***β*** **exon 3.** Sequences of MHC class II *β* among different species of Crocodylia, Aves and Mammalia are analysed using the amphibian sequence as an outgroup. Brackets on the right show vertebrate groups to which the MHC class II *β* sequences belong. Clades were defined on the basis of their monophyletic groupings and high posterior probabilities. Bootstrap values (BV) over 50 are provided on branches, and some below 50 are selectively present.
(PDF)

Figure S7 **Variable nucleotide sites of MHC class II** ***β*** **exon 3 sequences within Crocodylia.** Variable nucleotide positions are relative to the MHC class II *β* sequence from *C. rhombifer* (*Crrh-DB04*). Dots represent identical bases to the first sequence. The first column contains the names of MHC class II *β* sequences. Brackets on the right show Clades 1 and 2 of the MHC sequences, as have been explained in the text. Sites highlighted in red indicate fixed differences between Clade 1 and Clade 2 sequences.
(PDF)

Figure S8 **Distribution map of the Order Crocodylia showing the number of species per country.** This map does not show the actual distribution within each country, but the detailed distribution and list of species in each country can be obtained from http://crocodilian.com/cnhc/cnhc.html. This website also contains a list of primary references supporting this distribution map.
(PDF)

Table S1 **List of exon 3 sequences of MHC class II** ***β*** **across 20 species of Crocodylia investigated in the current studies (Up to two individuals per species studied).**
(PDF)

Table S2 **Summary of MHC class II** ***β*** **exon 3 sequences observed within a representative from each species of Crocodylia, where more than three sequences corresponding to at least two loci have been identified in this study.**
(PDF)

Table S3 **List of MHC class II** ***α*** **and** ***β*** **sequences available in GenBank used in three datasets for phylogenetic analyses of MHC class II sequences among major vertebrate classes.**
(PDF)

Appendix S1 **Characterisation of MHC class II** ***α*** **exons 2 and 3 within Crocodylia.**
(PDF)

Appendix S2 **Characterisation of MHC class II** ***β*** **exon 3 within Crocodylia.**
(PDF)

Appendix S3 **Effect of recombination at MHC class II** ***α*** **and** ***β.***
(PDF)

Appendix S4 **BLASTN searches of MHC class II primers used in this study against genome drafts of** ***Alligator mississippiensis*** **(v0.2.1),** ***Crocodylus porosus*** **(v0.2) and** ***Gangeticus gavialis*** **(v0.2).** These genome resources are available in GenBank and can be accessed with authors’ permission (St John et al. 2012). Good hits were observed between the primers and putative MHC class II sequences from those species, and this is likely to suggest an unbiased amplification of the presumed targets using the current primers. All the MHC class II sequences on the genomes were annotated and are expected to be published in another manuscript (Jaratlerdsiri et al. unpublished data).
(PDF)
