# Supplementary material for: Selection and Trans-Species Polymorphism of Major Histocompatibility Complex Class II Genes in the Order Crocodylia
Source: PLoS One. 2014 Feb 4;9(2):e87534. doi: 10.1371/journal.pone.0087534 (PMC3913596; doi:10.1371/journal.pone.0087534)
Supplement: Figure S1 — Amino acid alignments of MHC class II α sequences within Crocodylia. Variable positions are relative to the sequence at the top. The first column contains the names of MHC sequences from two families of Crocodylia: Crocodilidae (pale green colour) and Alligatoridae (pale red colour). The second column presents the amino acid alignment in letters. Dots represent amino acid identity to the top sequence; X letters represent unknown amino acids due to single-base deletions; asterisks represent stop codons; and numbers above the alignments represent the order of amino acid positions. Sites in boxes with closed triangles indicate conserved residues of antigen N and C termini on the peptide-binding region of the MHC class II α exon 2 alignment, as described in Kaufman et al. (1994); and sites in boxes linked with a line indicate the cysteine bridge (C-C) observed in the MHC class II α exon 3 alignment. Background colours in the alignments indicate degrees of amino acid identity: 100% in blue; 80–100% in yellow; and below 80% in white. The end of the alignments shows GenBank accession numbers. Trans-species polymorphisms (TSPs) are represented by numbers immediately after each MHC sequence. The same TSP is assigned with the same sequential number. (PDF) [file pone.0087534.s001.pdf]

Selection and trans-species polymorphism of Major Histocompatibility Complex class II genes in the Order Crocodylia

PLoS ONE

Weerachai Jaratlerdsiri[1], Sally R. Isberg[1,2], Damien P. Higgins[3], Lee G. Miles[1], Jaime Gongora[1,*]

[1] *Faculty of Veterinary Science, RMC Gunn Building, University of Sydney, Sydney, New South Wales 2006, Australia.*

[2] *Centre for Crocodile Research, P.O. Box 329, Noonamah, Northern Territory 0837, Australia.*

[3] *Faculty of Veterinary Science, McMaster Building, University of Sydney, New South Wales 2006, Australia.*

* Corresponding author: Phone: +61-2 9036 9348. Fax: +61-2 9351 3957. E-mail: jaime.gongora@sydney.edu.au

MHC class II *α* exon 2

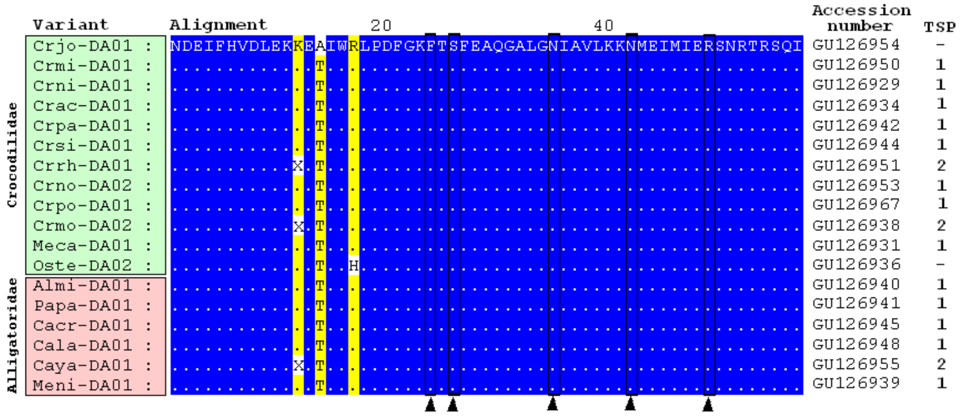

MHC class II *α* exon 3

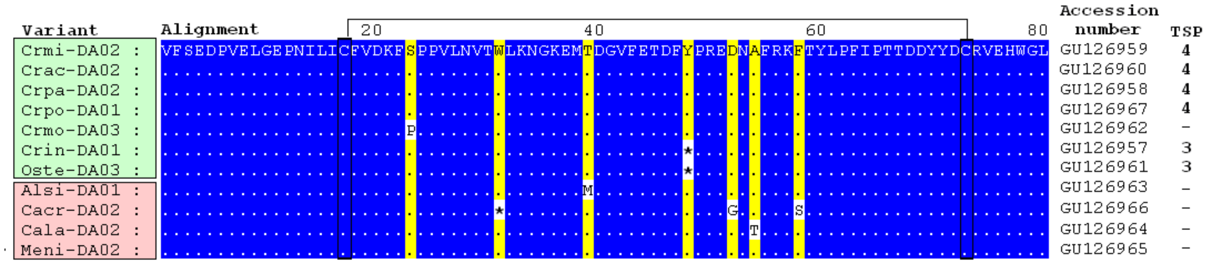

Figure S1. Amino acid alignments of MHC class II *α* sequences within Crocodylia. Variable positions are relative to the sequence at the top. The first column contains the names of MHC sequences from two families of Crocodylia: Crocodilidae (pale green colour) and Alligatoridae (pale red colour). The second column presents the amino acid alignment in letters. Dots represent amino acid identity to the top sequence; X letters represent unknown amino acids due to single-base deletions; asterisks represent stop codons; and numbers above the alignments represent the order of amino acid positions. Sites in boxes with closed triangles indicate conserved residues of antigen N and C termini on the peptide-binding region of the MHC class II *α* exon 2 alignment, as described in Kaufman et al (1994); and sites in boxes linked with a line indicate the cysteine bridge (C-C) observed in the MHC class II *α* exon 3 alignment. Background colours in the alignments indicate degrees of amino acid identity: 100% in blue; 80-100% in yellow; and below 80% in white. The end of the alignments shows GenBank accession numbers. Trans-species polymorphisms (TSPs) are represented by numbers immediately after each MHC sequence. The same TSP is assigned with the same sequential number
