# Supplementary material for: Selection and Trans-Species Polymorphism of Major Histocompatibility Complex Class II Genes in the Order Crocodylia
Source: PLoS One. 2014 Feb 4;9(2):e87534. doi: 10.1371/journal.pone.0087534 (PMC3913596; doi:10.1371/journal.pone.0087534)
Supplement: Figure S3 — Amino acid alignment of MHC class II β exons 2 and 3 used for selection detection tests. The first column contains the names of MHC sequences. The second column presents the amino acid alignment in letters. Question marks represent unknown amino acids, and numbers above the alignments represent the order of amino acid positions. Sites in boxes with open triangles indicate potential peptide contact residues on the peptide binding region of the HLA-DRB1 molecule based on crystallography models (Bondinas et al. 2007), and those in boxes with closed triangles indicate conserved residues of antigen N and C termini on the peptide-binding region of the MHC class II β molecule (Kaufman et al. 1994). (PDF) [file pone.0087534.s003.pdf]

Selection and trans-species polymorphism of Major Histocompatibility Complex class II genes in the Order Crocodylia

PLoS ONE

Weerachai Jaratlerdsiri[1], Sally R. Isberg[1,2], Damien P. Higgins[3], Lee G. Miles[1], Jaime Gongora[1,*]

[1] *Faculty of Veterinary Science, RMC Gunn Building, University of Sydney, Sydney, New South Wales 2006, Australia.*

[2] *Centre for Crocodile Research, P.O. Box 329, Noonamah, Northern Territory 0837, Australia.*

[3] *Faculty of Veterinary Science, McMaster Building, University of Sydney, New South Wales 2006, Australia.*

* Corresponding author: Phone: +61-2 9036 9348. Fax: +61-2 9351 3957. E-mail: jaime.gongora@sydney.edu.au

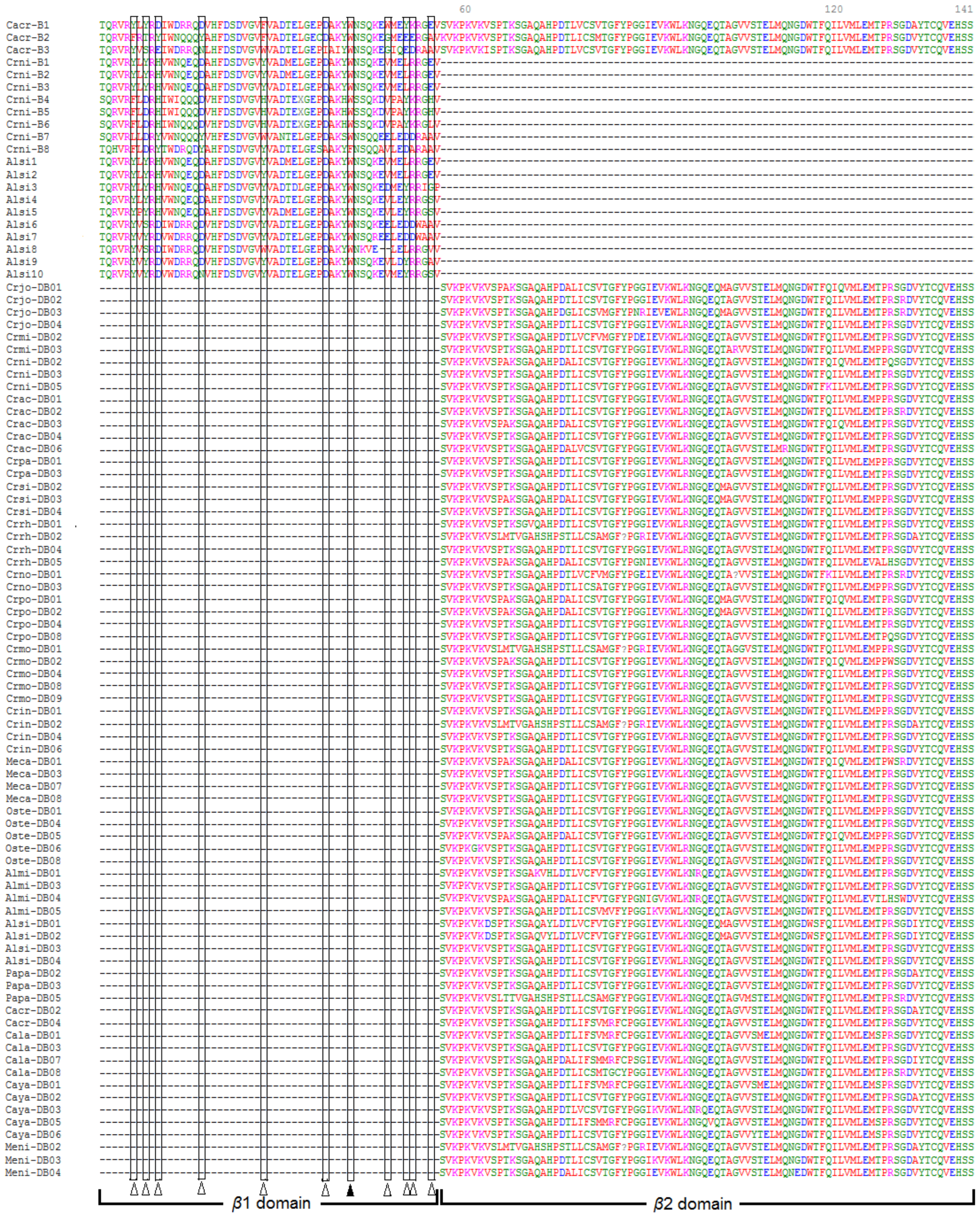

Figure S3. Amino acid alignment of MHC class II *β* exons 2 and 3 used for selection detection tests. The first column contains the names of MHC sequences. The second column presents the amino acid alignment in letters. Question marks represent unknown amino acids, and numbers above the alignments represent the order of amino acid positions. Sites in boxes with open triangles indicate potential peptide contact residues on the peptide binding

region of the HLA-DRB1 molecule based on crystallography models (Bondinas et al. 2007), and those in boxes with closed triangles indicate conserved residues of antigen N and C termini on the peptide-binding region of the MHC class II β molecule (Kaufman et al. 1994)
