# Supplementary material for: Selection and Trans-Species Polymorphism of Major Histocompatibility Complex Class II Genes in the Order Crocodylia
Source: PLoS One. 2014 Feb 4;9(2):e87534. doi: 10.1371/journal.pone.0087534 (PMC3913596; doi:10.1371/journal.pone.0087534)
Supplement: Figure S5 — Maximum-likelihood trees of (A) MHC class II α exon 2, and (B) exon 3. Sequences of MHC class II α among different species of Crocodylia, birds and mammals are analysed using the shark Gici sequences as an outgroup. Brackets in the middle show vertebrate groups to which the MHC class II α sequences belong. Bootstrap values (BV) over 50 are provided on branches. ELW tests show that these two trees did not differ significantly from their Bayesian trees of MHC class II α exon 2 sequences described in Fig. 2A (confidence tree set = 0.79 and 0.21), and MHC class II α exon 3 sequences described in Fig. 2B (confidence tree set = 0.62 and 0.38). (PDF) [file pone.0087534.s005.pdf]

Selection and trans-species polymorphism of Major Histocompatibility Complex class II genes in the Order Crocodylia

PLoS ONE

Weerachai Jaratlerdsiri[1], Sally R. Isberg[1,2], Damien P. Higgins[3], Lee G. Miles[1], Jaime Gongora[1,*]

[1] *Faculty of Veterinary Science, RMC Gunn Building, University of Sydney, Sydney, New South Wales 2006, Australia.*

[2] *Centre for Crocodile Research, P.O. Box 329, Noonamah, Northern Territory 0837, Australia.*

[3] *Faculty of Veterinary Science, McMaster Building, University of Sydney, New South Wales 2006, Australia.*

* Corresponding author: Phone: +61-2 9036 9348. Fax: +61-2 9351 3957. E-mail: jaime.gongora@sydney.edu.au

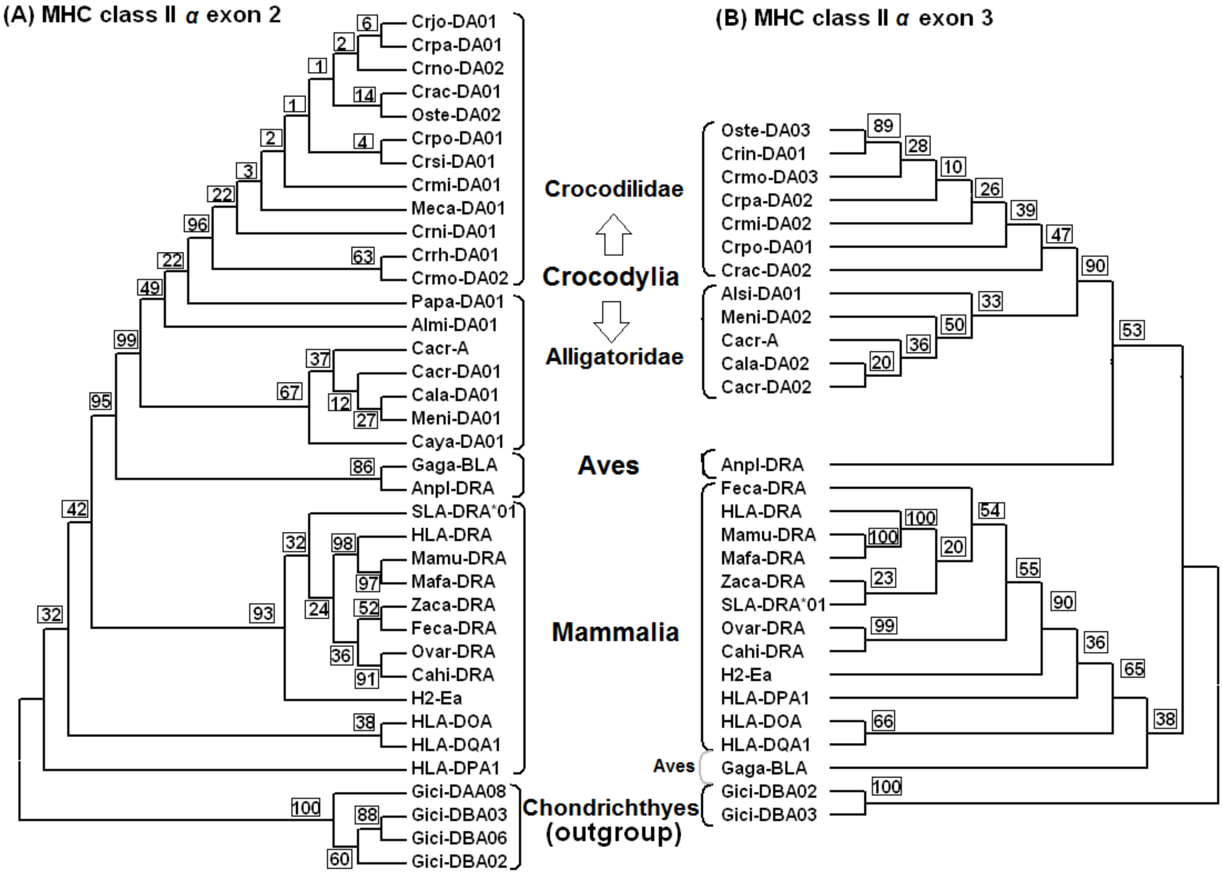

Figure S5. Maximum-likelihood trees of (A) MHC class II *α* exon 2, and (B) exon 3. Sequences of MHC class II *α* among different species of Crocodylia, birds and mammals are analysed using the shark *Gici* sequences as an outgroup. Brackets in the middle show vertebrate groups to which the MHC class II *α* sequences belong. Bootstrap values (BV) over 50 are provided on branches. ELW tests show that these two trees did not differ significantly from their Bayesian trees of MHC class II *α* exon 2 sequences described in Fig. 2A (confidence tree set = 0.79 and 0.21), and MHC class II *α* exon 3 sequences described in Fig. 2B (confidence tree set = 0.62 and 0.38)
