# Supplementary material for: Selection and Trans-Species Polymorphism of Major Histocompatibility Complex Class II Genes in the Order Crocodylia
Source: PLoS One. 2014 Feb 4;9(2):e87534. doi: 10.1371/journal.pone.0087534 (PMC3913596; doi:10.1371/journal.pone.0087534)
Supplement: Figure S6 — Maximum-likelihood analysis of MHC class II β exon 3. Sequences of MHC class II β among different species of Crocodylia, Aves and Mammalia are analysed using the amphibian sequence as an outgroup. Brackets on the right show vertebrate groups to which the MHC class II β sequences belong. Clades were defined on the basis of their monophyletic groupings and high posterior probabilities. Bootstrap values (BV) over 50 are provided on branches, and some below 50 are selectively present. (PDF) [file pone.0087534.s006.pdf]

Selection and trans-species polymorphism of Major Histocompatibility Complex class II genes in the Order Crocodylia

PLoS ONE

Weerachai Jaratlerdsiri[1], Sally R. Isberg[1,2], Damien P. Higgins[3], Lee G. Miles[1], Jaime Gongora[1,*]

[1] *Faculty of Veterinary Science, RMC Gunn Building, University of Sydney, Sydney, New South Wales 2006, Australia.*

[2] *Centre for Crocodile Research, P.O. Box 329, Noonamah, Northern Territory 0837, Australia.*

[3] *Faculty of Veterinary Science, McMaster Building, University of Sydney, New South Wales 2006, Australia.*

* Corresponding author: Phone: +61-2 9036 9348. Fax: +61-2 9351 3957. E-mail: jaime.gongora@sydney.edu.au

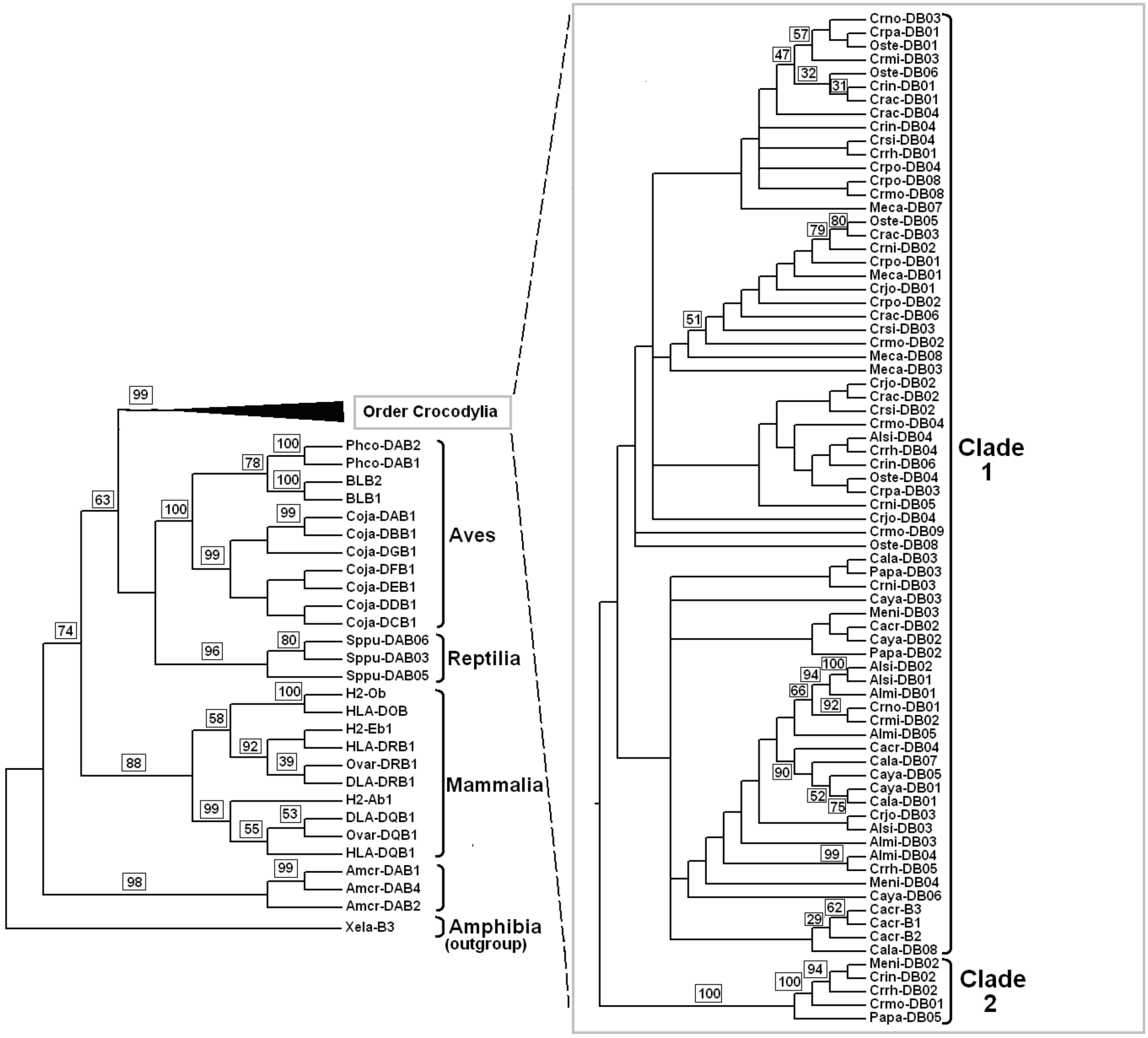

Figure S6. Maximum-likelihood analysis of MHC class II *β* exon 3. Sequences of MHC class II *β* among different species of Crocodylia, Aves and Mammalia are analysed using the amphibian sequence as an outgroup. Brackets on the right show vertebrate groups to which the MHC class II *β* sequences belong. Clades were defined on the basis of their monophyletic groupings and high posterior probabilities. Bootstrap values (BV) over 50 are provided on branches, and some below 50 are selectively present
