# Supplementary material for: Selection and Trans-Species Polymorphism of Major Histocompatibility Complex Class II Genes in the Order Crocodylia
Source: PLoS One. 2014 Feb 4;9(2):e87534. doi: 10.1371/journal.pone.0087534 (PMC3913596; doi:10.1371/journal.pone.0087534)
Supplement: Figure S7 — Variable nucleotide sites of MHC class II β exon 3 sequences within Crocodylia. Variable nucleotide positions are relative to the MHC class II β sequence from C. rhombifer (Crrh-DB04). Dots represent identical bases to the first sequence. The first column contains the names of MHC class II β sequences. Brackets on the right show Clades 1 and 2 of the MHC sequences, as have been explained in the text. Sites highlighted in red indicate fixed differences between Clade 1 and Clade 2 sequences. (PDF) [file pone.0087534.s007.pdf]

Selection and trans-species polymorphism of Major Histocompatibility Complex class II genes in the Order Crocodylia

PLoS ONE

Weerachai Jaratlerdsiri[1], Sally R. Isberg[1,2], Damien P. Higgins[3], Lee G. Miles[1], Jaime Gongora[1,*]

[1] *Faculty of Veterinary Science, RMC Gunn Building, University of Sydney, Sydney, New South Wales 2006, Australia.*

[2] *Centre for Crocodile Research, P.O. Box 329, Noonamah, Northern Territory 0837, Australia.*

[3] *Faculty of Veterinary Science, McMaster Building, University of Sydney, New South Wales 2006, Australia.*

* Corresponding author: Phone: +61-2 9036 9348. Fax: +61-2 9351 3957. E-mail: jaime.gongora@sydney.edu.au

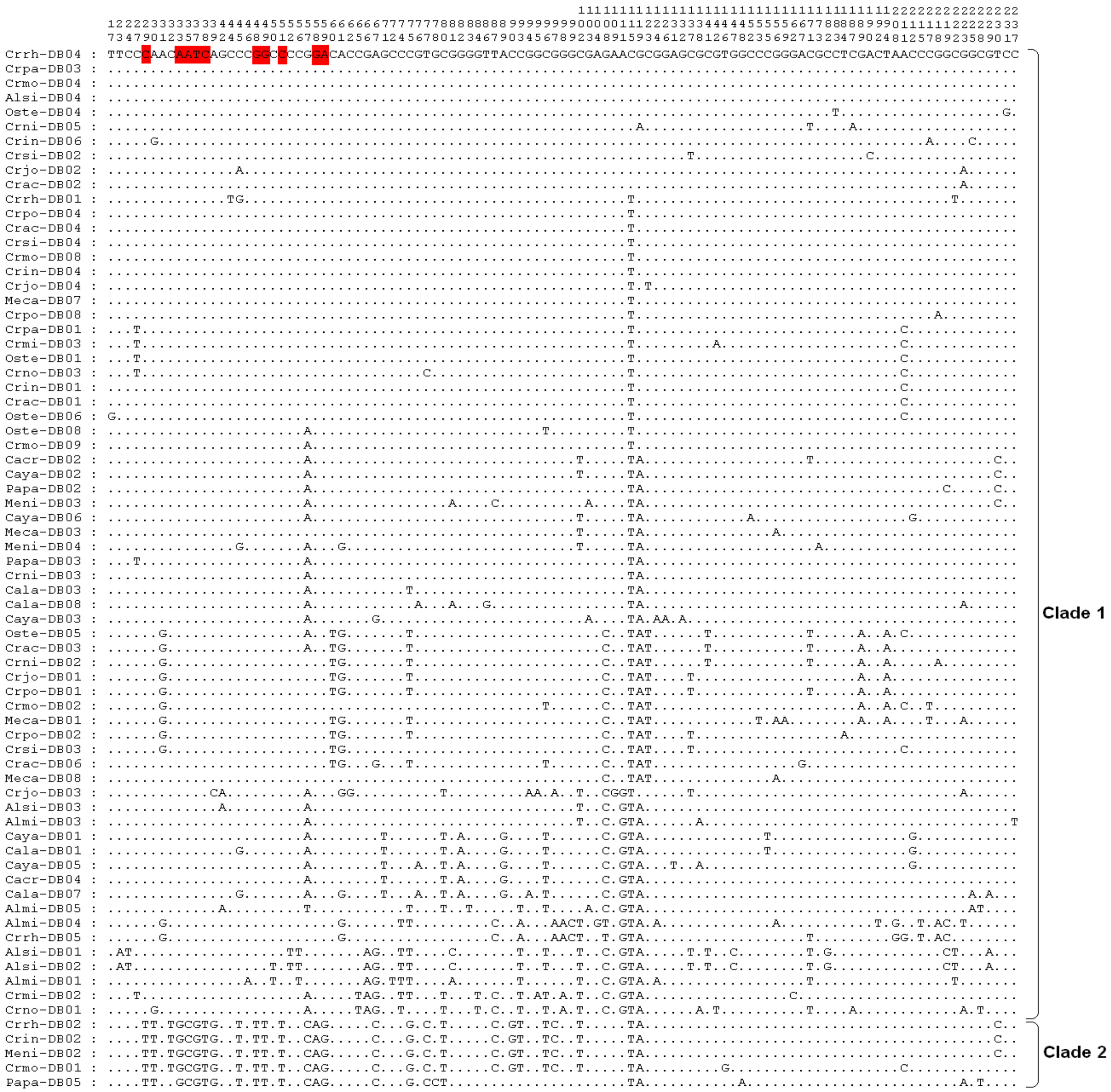

Figure S7. Variable nucleotide sites of MHC class II *β* exon 3 sequences within Crocodylia. Variable nucleotide positions are relative to the MHC class II *β* sequence from *C. rhombifer* (*Crrh-DB04*). Dots represent identical bases to the first sequence. The first column contains the names of MHC class II *β* sequences. Brackets on the right show Clades 1 and 2 of the MHC sequences, as have been explained in the text. Sites highlighted in red indicate fixed differences between Clade 1 and Clade 2 sequences
