# Supplementary material for: Selection and Trans-Species Polymorphism of Major Histocompatibility Complex Class II Genes in the Order Crocodylia
Source: PLoS One. 2014 Feb 4;9(2):e87534. doi: 10.1371/journal.pone.0087534 (PMC3913596; doi:10.1371/journal.pone.0087534)
Supplement: Figure S8 — Distribution map of the Order Crocodylia showing the number of species per country. This map does not show the actual distribution within each country, but the detailed distribution and list of species in each country can be obtained from http://crocodilian.com/cnhc/cnhc.html. This website also contains a list of primary references supporting this distribution map. (PDF) [file pone.0087534.s008.pdf]

Selection and trans-species polymorphism of Major Histocompatibility Complex class II genes in the Order Crocodylia

PLoS ONE

Weerachai Jaratlerdsiri[1], Sally R. Isberg[1,2], Damien P. Higgins[3], Lee G. Miles[1], Jaime Gongora[1,*]

[1] *Faculty of Veterinary Science, RMC Gunn Building, University of Sydney, Sydney, New South Wales 2006, Australia.*

[2] *Centre for Crocodile Research, P.O. Box 329, Noonamah, Northern Territory 0837, Australia.*

[3] *Faculty of Veterinary Science, McMaster Building, University of Sydney, New South Wales 2006, Australia.*

* Corresponding author: Phone: +61-2 9036 9348. Fax: +61-2 9351 3957. E-mail: jaime.gongora@sydney.edu.au

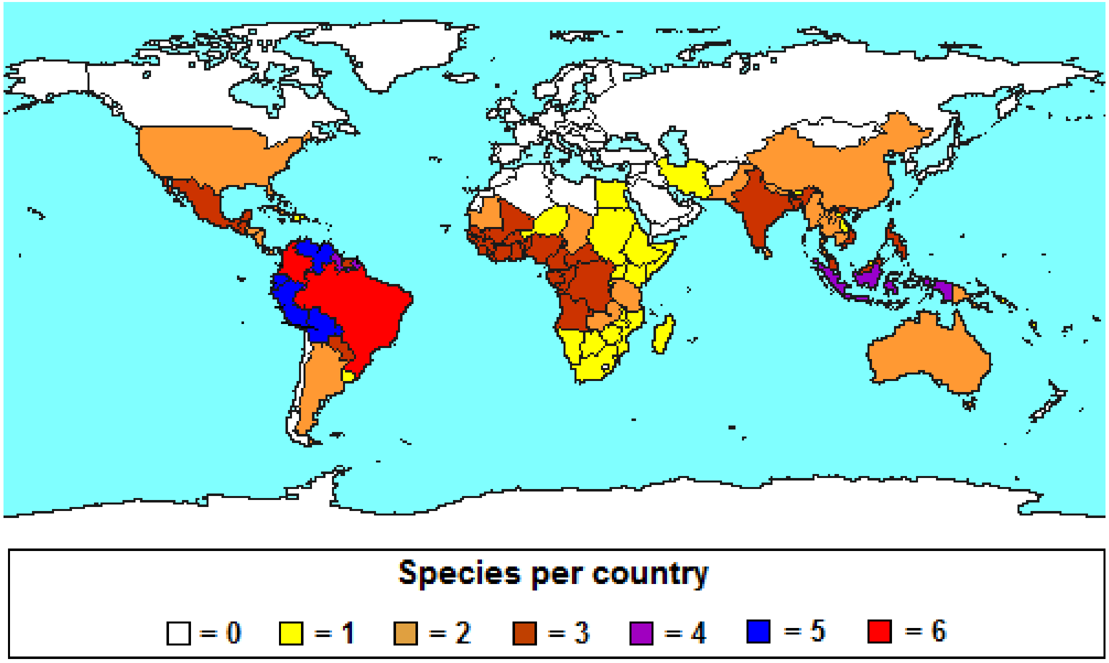

Figure S8. Distribution map of the Order Crocodylia showing the number of species per country. This map does not show the actual distribution within each country, but the detailed distribution and list of species in each country can be obtained from http://crocodilian.com/cnhc/cnhc.html. This website also contains a list of primary references supporting this distribution map
