# Supplementary material for: Selection and Trans-Species Polymorphism of Major Histocompatibility Complex Class II Genes in the Order Crocodylia
Source: PLoS One. 2014 Feb 4;9(2):e87534. doi: 10.1371/journal.pone.0087534 (PMC3913596; doi:10.1371/journal.pone.0087534)
Supplement: Table S1 — List of exon 3 sequences of MHC class II β across 20 species of Crocodylia investigated in the current studies (Up to two individuals per species studied). (PDF) [file pone.0087534.s009.pdf]

Selection and trans-species polymorphism of Major Histocompatibility Complex class II genes in the Order Crocodylia

PLoS ONE

Weerachai Jaratlerdsiri[1], Sally R. Isberg[1,2], Damien P. Higgins[3], Lee G. Miles[1], Jaime Gongora[1,*]

[1] *Faculty of Veterinary Science, RMC Gunn Building, University of Sydney, Sydney, New South Wales 2006, Australia.*

[2] *Centre for Crocodile Research, P.O. Box 329, Noonamah, Northern Territory 0837, Australia.*

[3] *Faculty of Veterinary Science, McMaster Building, University of Sydney, New South Wales 2006, Australia.*

* Corresponding author: Phone: +61-2 9036 9348. Fax: +61-2 9351 3957. E-mail: jaime.gongora@sydney.edu.au

Table S1. List of exon 3 sequences of MHC class II β across 20 species of Crocodylia investigated in the current studies (Up to two individuals per species studied)

Family	Species	MHC class II β exon 3 sequence	
		**Individual 1**	**Individual 2**
Crocodilidae	*Crocodylus johnsoni*	*Crjo-DB01, 03 & 04*	*Crjo-DB02*
	Crocodylus mindorensis	*Crmi-DB02 & 03*	NA[a]
	Crocodylus niloticus	*Crni-DB02 & 03*	*Crni-DB05*
	Crocodylus acutus	*Crac-DB01 & 03*	*Crac-DB02, 04 & 06*
	Crocodylus palustris	*Crpa-DB01 & 03*	NA
	Osteolaemus tetraspis	*Oste-DB01, 04, 05 & 08*	*Oste-DB06*
	Crocodylus siamensis	*Crsi-DB02–04*	*Crsi-DB04*
	Mecistops cataphractus	*Meca-DB01, 03, 07 & 08*	*Meca-DB07*
	Crocodylus intermedius	*Crin-DB01 & 02* (ψ[b])	*Crin-DB04 & 06*
	Crocodylus rhombifer	*Crrh-DB01, 04 & 05*	*Crrh-DB02* (ψ)
	Crocodylus novaeguineae	*Crno-DB01* (ψ) *& 03*	NA
	Crocodylus porosus	*Crpo-DB01, 02 & 04*	*Crpo-DB08*
	Crocodylus moreletii	*Crmo-DB01* (ψ) *& 02*	*Crmo-DB04, 08 & 09*
Alligatoridae	*Alligator mississippiensis*	*Almi-DB01, 04 & 05*	*Almi-DB03*
	Alligator sinensis	*Alsi-DB01, 02 & 04*	*Alsi-DB02 & 03*
	Paleosuchus palpebrosus	NA	*Papa-DB02, 03, & 05*
	Caiman crocodylus	NA	*Cacr-DB02 & 04*
	Caiman latirostris	*Cala-DB01, 03 & 08*	*Cala-DB07*
	Caiman yacare	*Caya-DB01 & 03*	*Caya-DB02, 05 & 06*
	Melanosuchus niger	*Meni-DB02* (ψ)	*Meni-DB02* (ψ)*-04*

[a] Not applicable
[b] putative pseudogenes
