# Supplementary material for: Selection and Trans-Species Polymorphism of Major Histocompatibility Complex Class II Genes in the Order Crocodylia
Source: PLoS One. 2014 Feb 4;9(2):e87534. doi: 10.1371/journal.pone.0087534 (PMC3913596; doi:10.1371/journal.pone.0087534)
Supplement: Table S2 — Summary of MHC class II β exon 3 sequences observed within a representative from each species of Crocodylia, where more than three sequences corresponding to at least two loci have been identified in this study. (PDF) [file pone.0087534.s010.pdf]

Selection and trans-species polymorphism of Major Histocompatibility Complex class II genes in the Order Crocodylia

PLoS ONE

Weerachai Jaratlerdsiri[1], Sally R. Isberg[1,2], Damien P. Higgins[3], Lee G. Miles[1], Jaime Gongora[1,*]

[1] *Faculty of Veterinary Science, RMC Gunn Building, University of Sydney, Sydney, New South Wales 2006, Australia.*

[2] *Centre for Crocodile Research, P.O. Box 329, Noonamah, Northern Territory 0837, Australia.*

[3] *Faculty of Veterinary Science, McMaster Building, University of Sydney, New South Wales 2006, Australia.*

* Corresponding author: Phone: +61-2 9036 9348. Fax: +61-2 9351 3957. E-mail: jaime.gongora@sydney.edu.au

Table S2. Summary of MHC class II β exon 3 sequences observed within a representative from each species of Crocodylia, where more than three sequences corresponding to at least two loci have been identified in this study

Species	Sequences/ ind.[a]	Clade 1[b]							Clade 2[b]
		1A	1B	1C	1D	1E	1F	1G	
1. *C. johnsoni*	3	1		1	1				
2. *C. acutus*	3	2		1					
3. *O. tetraspis*	4	3		1					
4. *C. siamensis*	3	2		1					
5. *M. cataphractus*	4	1	1	2					
6. *C. rhombifer*	3	2					1		
7. *C. porosus*	3	1		2					
8. *C. moreletii*	3	3							
9. *A. mississippiensis*	3					1	1	1	
10. *A. sinensis*	3	1						2	
11. *P. palpebrosus*	3		2						1
12. *C. latirostris*	3		2			1			
13. *C. yacare*	3		2			1			
14. *M. niger*	3		2						1 (ψ[c])

[a] Number of MHC class II β exon 3 sequences per individual

[b] Filled areas with number indicate clades (1 and 2) or subclades (1A-1G) that are found to cluster certain number of MHC sequence(s) from each species of Crocodylia studied

[c] ψ indicates a putative pseudogene
