# Supplementary material for: Selection and Trans-Species Polymorphism of Major Histocompatibility Complex Class II Genes in the Order Crocodylia
Source: PLoS One. 2014 Feb 4;9(2):e87534. doi: 10.1371/journal.pone.0087534 (PMC3913596; doi:10.1371/journal.pone.0087534)
Supplement: Table S3 — List of MHC class II α and β sequences available in GenBank used in three datasets for phylogenetic analyses of MHC class II sequences among major vertebrate classes. (PDF) [file pone.0087534.s011.pdf]

Selection and trans-species polymorphism of Major Histocompatibility Complex class II genes in the Order Crocodylia

PLoS ONE

Weerachai Jaratlerdsiri[1], Sally R. Isberg[1,2], Damien P. Higgins[3], Lee G. Miles[1], Jaime Gongora[1,*]

[1] *Faculty of Veterinary Science, RMC Gunn Building, University of Sydney, Sydney, New South Wales 2006, Australia.*

[2] *Centre for Crocodile Research, P.O. Box 329, Noonamah, Northern Territory 0837, Australia.*

[3] *Faculty of Veterinary Science, McMaster Building, University of Sydney, New South Wales 2006, Australia.*

* Corresponding author: Phone: +61-2 9036 9348. Fax: +61-2 9351 3957. E-mail: jaime.gongora@sydney.edu.au

Table S3. List of MHC class II α and β sequences available in GenBank used in three datasets for phylogenetic analyses of MHC class II sequences among major vertebrate classes

Dataset	Class	Species	Sequence name	Accession no.
MHC class II α exons 2 and 3	Reptilia	*Caiman crocodilus* (caiman)	*Cacr-A*	AF256650
	Aves	*Gallus gallus* (chicken)	*Gaga-BLA*	AY357253
		Anas platyrhynchos (mallard)	*Anpl-DRA*	AY905539
	Mammalia	*Felis catus* (domestic cat)	*Feca-DRA*	EU915361
		Zalophus californianus (sea lion)	*Zaca-DRA*	AY491455
		Sus scrofa (pig)	*SLA-DRA*01*	DQ883224
		Capra hircus (goat)	*Cahi-DRA*	AB008754
		Ovis aries (sheep)	*Ovar-DRA*	FM986335
		Macaca fascicularis (macaque)	*Mafa-DRA*	AB306651
		Macaca mulatta (rhesus monkey)	*Mamu-DRA*	NM_001134298
		Mus musculus (mouse)	*H2-Ea*	BC106107
		Homo sapiens (human)	*HLA-DRA*	NM_019111
			HLA-DQA1	NM_002122
			HLA-DOA	NM_002119
			HLA-DPA1	NM_033554
	Chondrichthyes (outgroup)	*Ginglymostoma cirratum* (shark)	*Gici-DBA03*	AF220357
			Gici-DBA02	AF220356
			Gici-DBA06	AF220224
			Gici-DAA08	AF220223
MHC class II β exon 3	Reptilia	*Caiman crocodilus* (caiman)	*Cacr-B1*	AF256651
			Cacr-B2	AF256652
			Cacr-B3	AF277661
		Sphenodon punctatus (tuatara)	*Sppu-DAB*03*	DQ124235
			*Sppu-DAB*05*	DQ124237
			*Sppu-DAB*06*	DQ124238
		Amblyrhynchus cristatus (iguana)	*Amcr-DAB1*	FJ623746
			Amcr-DAB2	FJ623747
			Amcr-DAB4	FJ623751
	Aves	*Gallus gallus* (chicken)	*BLB1*	AL023516
			BLB2	
		Coturnix japonica (quail)	*Coja-DAB1*	AB078884
			Coja-DBB1	
			Coja-DCB1	
			Coja-DDB1	
			Coja-DEB1	
			Coja-DFB1	
			Coja-DGB1	
		Phasianus colchicus (pheasant)	*Phco-DAB1*	AJ224349
			Phco-DAB2	AJ224348
	Mammalia	*Canis lupus familiaris* (dog)	*DLA-DRB1*	NM_001014768
			DLA-DQB1	NM_001014381
		Ovis aries (sheep)	*Ovar-DRB1*	NM_001123402
			Ovar-DQB1	L08792
		Mus musculus (mouse)	*H2-Eb1*	NM_010382
			H2-Ab1	NM_207105
			H2-Ob	NM_ 010389
		Homo sapiens (human)	*HLA-DQB1*	NM_002123
			HLA-DOB	NM_002120
			HLA-DRB1	NM_002124
	Amphibia (outgroup)	*Xenopus laevis* (frog)	*Xela-B3*	D13685
