# Supplementary material for: Selection and Trans-Species Polymorphism of Major Histocompatibility Complex Class II Genes in the Order Crocodylia
Source: PLoS One. 2014 Feb 4;9(2):e87534. doi: 10.1371/journal.pone.0087534 (PMC3913596; doi:10.1371/journal.pone.0087534)
Supplement: Appendix S1 — Characterisation of MHC class II α exons 2 and 3 within Crocodylia. (PDF) [file pone.0087534.s012.pdf]

Selection and trans-species polymorphism of Major Histocompatibility Complex class II genes in the Order Crocodylia

PLoS ONE

Weerachai Jaratlerdsiri[1], Sally R. Isberg[1,2], Damien P. Higgins[3], Lee G. Miles[1], Jaime Gongora[1,*]

[1] *Faculty of Veterinary Science, RMC Gunn Building, University of Sydney, Sydney, New South Wales 2006, Australia.*

[2] *Centre for Crocodile Research, P.O. Box 329, Noonamah, Northern Territory 0837, Australia.*

[3] *Faculty of Veterinary Science, McMaster Building, University of Sydney, New South Wales 2006, Australia.*

* Corresponding author: Phone: +61-2 9036 9348. Fax: +61-2 9351 3957. E-mail: jaime.gongora@sydney.edu.au

Appendix S1. Characterisation of MHC class II α exons 2 and 3 within Crocodylia

Eighteen and eleven sequences of MHC class II α exons 2 and 3 respectively were identified among species of Crocodylia (Table 1; Figure S1). Single sequences per specimen and species were retrieved for both of these exons. It could be interpreted as a single locus was amplified. However, likelihood of additional gene copy number within these species cannot be excluded. All MHC class II α exon 2 sequences had 171 bp in length, except for three sequences containing 170 bp in length due to a single-base deletion at base 34, while an expected amplicon size (240 bp) was obtained in the MHC class II α exon 3. For MHC class II α exon 2, six polymorphic nucleotide sites (3.51% of the sequence length) were identified in the sequence alignment, while 18 polymorphic sites (7.92%) for MHC class II α exon 3. Four of these sites in exon 2 were synonymous substitutions and nine in exon 3. Pairwise differences between the exon 2 sequences revealed 1.64 substitutions on average (range 0.93-2.35 substitutions), while pairwise differences in exon 3 showed 5.31 substitutions on average (range 4.11-6.51 substitutions). For the nine species of Crocodylia in which both exons 2 and 3 were characterised successfully (Table 1), relatively low numbers of polymorphic sites and high percentage of synonymous change were still consistent with the comparison between all the MHC class II α sequences described above.
