# Supplementary material for: Selection and Trans-Species Polymorphism of Major Histocompatibility Complex Class II Genes in the Order Crocodylia
Source: PLoS One. 2014 Feb 4;9(2):e87534. doi: 10.1371/journal.pone.0087534 (PMC3913596; doi:10.1371/journal.pone.0087534)
Supplement: Appendix S2 — Characterisation of MHC class II β exon 3 within Crocodylia. (PDF) [file pone.0087534.s013.pdf]

Selection and trans-species polymorphism of Major Histocompatibility Complex class II genes in the Order Crocodylia

PLoS ONE

Weerachai Jaratlerdsiri[1], Sally R. Isberg[1,2], Damien P. Higgins[3], Lee G. Miles[1], Jaime Gongora[1,*]

[1] *Faculty of Veterinary Science, RMC Gunn Building, University of Sydney, Sydney, New South Wales 2006, Australia.*

[2] *Centre for Crocodile Research, P.O. Box 329, Noonamah, Northern Territory 0837, Australia.*

[3] *Faculty of Veterinary Science, McMaster Building, University of Sydney, New South Wales 2006, Australia.*

* Corresponding author: Phone: +61-2 9036 9348. Fax: +61-2 9351 3957. E-mail: jaime.gongora@sydney.edu.au

Appendix S2. Characterisation of MHC class II β exon 3 within Crocodylia

Seventy-two sequences (260 bp) of MHC class II β exon 3 were identified among 20 species of Crocodylia (Table 1; Figure S2). Between one to four sequences per individual within a species were found suggesting that at least two loci were being amplified in the current study. In total, 70 variable nucleotide substitutions corresponding to 26.92% of the sequence analysed were identified across the sequence alignment of the 72 sequences. Unexpectedly, low number of nucleotide substitutions was observed at a $CD4^+$ binding site between bases 124-198. Thirty-three out of 70 substitutions were synonymous, while 37 were nonsynonymous substitutions. Among-species pairwise differences between the MHC class II β sequences showed 13.67 substitutions on average (range 12.23-15.11 substitutions).
