# Supplementary material for: Selection and Trans-Species Polymorphism of Major Histocompatibility Complex Class II Genes in the Order Crocodylia
Source: PLoS One. 2014 Feb 4;9(2):e87534. doi: 10.1371/journal.pone.0087534 (PMC3913596; doi:10.1371/journal.pone.0087534)
Supplement: Appendix S3 — Effect of recombination at MHC class II α and β. (PDF) [file pone.0087534.s014.pdf]

Selection and trans-species polymorphism of Major Histocompatibility Complex class II genes in the Order Crocodylia

PLoS ONE

Weerachai Jaratlerdsiri[1], Sally R. Isberg[1,2], Damien P. Higgins[3], Lee G. Miles[1], Jaime Gongora[1,*]

[1] *Faculty of Veterinary Science, RMC Gunn Building, University of Sydney, Sydney, New South Wales 2006, Australia.*

[2] *Centre for Crocodile Research, P.O. Box 329, Noonamah, Northern Territory 0837, Australia.*

[3] *Faculty of Veterinary Science, McMaster Building, University of Sydney, New South Wales 2006, Australia.*

* Corresponding author: Phone: +61-2 9036 9348. Fax: +61-2 9351 3957. E-mail: jaime.gongora@sydney.edu.au

Appendix S3. Effect of recombination at MHC class II *α* and *β*

Three recombinants from three species of Crocodilidae (*Crmo-DB01*, *Crrh-DB02*, and *Crin-DB02*) and two from two species of Alligatoridae (*Meni-DB02* and *Papa-DB05*) were identified among all the 72 MHC class II *β* exon 3 sequences, while none were observed among MHC class II *α* exons 2 and 3 sequences (Table A). The MHC class II *α* sequences from extant species of Crocodylia also showed almost four times lower mean amount of recombination rate per amino acid site (ρ = 0.027) than the mean number of nucleotide substitutions among the sequences (θ = 0.1) using omegaMap (Figure A-A). Similarly, the mean ρ value among the MHC class II *β* sequences, which was equal to 0.267 per amino acid was lower than the θ value of 0.475 (Figure A-B). These supports that recombination does not play a major role in generating diversity among the MHC class II sequences identified in the current study.

Table A. Recombinant sequences and their attributes, including consensus scores, expected parental sequences, and length of recombining segments between the parental sequences

Recombinant sequence[a]	Consensus Score	Potential parental sequence	Length of recombining segment
Crmo-DB01	70	*Crsi-DB03*, unknown	MHC class II B exon 3 (149 bp)
Crrh-DB02	70	*Meca-DB01*, unknown	MHC class II B exon 3 (258 bp)
Crin-DB02	70	*Meca-DB01*, unknown	MHC class II B exon 3 (258 bp)
Meni-DB02	70	*Meca-DB01*, unknown	MHC class II B exon 3 (258 bp)
Papa-DB05	60	*Crac-DB02*, unknown	MHC class II B exon 3 (165 bp)

[a] Recombination detected by more than one of eight algorithms, that is, RDP, Genconv, Chimeara, Maxchi, SiScan, 3seq, LARD and Bootscan

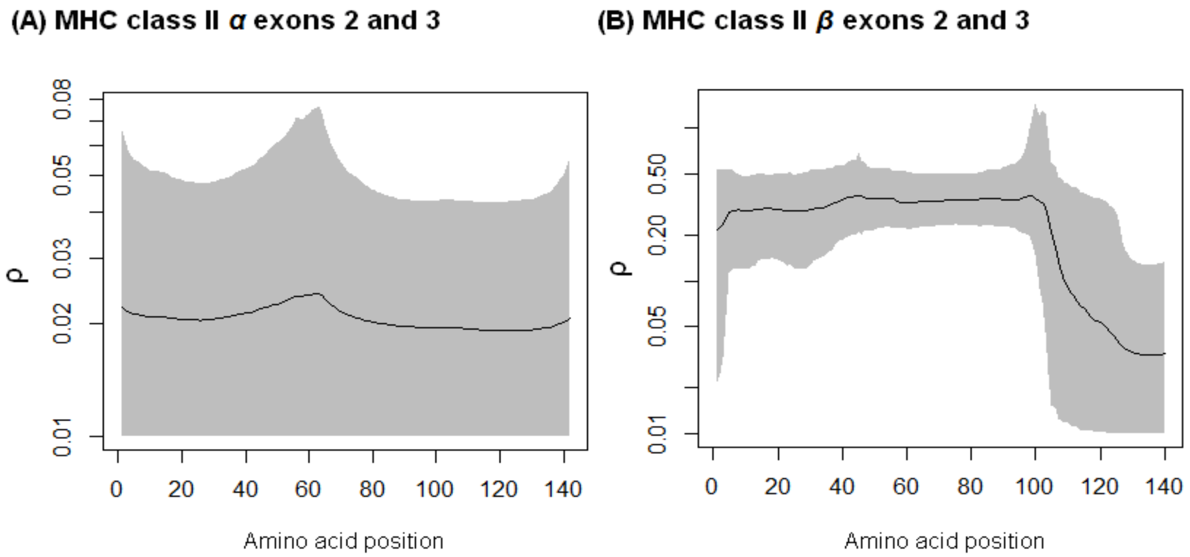

Figure A. Recombination rate across amino acids of (A) MHC class II *α* and (B) II *β*. Lines indicate a mean estimate of this rate. Grey areas represent 95% highest posterior probability dense intervals
