# Supplementary material for: Selection and Trans-Species Polymorphism of Major Histocompatibility Complex Class II Genes in the Order Crocodylia
Source: PLoS One. 2014 Feb 4;9(2):e87534. doi: 10.1371/journal.pone.0087534 (PMC3913596; doi:10.1371/journal.pone.0087534)
Supplement: Appendix S4 — BLASTN searches of MHC class II primers used in this study against genome drafts of Alligator mississippiensis (v0.2.1), Crocodylus porosus (v0.2) and Gangeticus gavialis (v0.2). These genome resources are available in GenBank and can be accessed with authors’ permission (St John et al. 2012). Good hits were observed between the primers and putative MHC class II sequences from those species, and this is likely to suggest an unbiased amplification of the presumed targets using the current primers. All the MHC class II sequences on the genomes were annotated and are expected to be published in another manuscript (Jaratlerdsiri et al. unpublished data). (PDF) [file pone.0087534.s015.pdf]

Selection and trans-species polymorphism of Major Histocompatibility Complex class II genes in the Order Crocodylia

PLoS ONE

Weerachai Jaratlerdsiri[1], Sally R. Isberg[1,2], Damien P. Higgins[3], Lee G. Miles[1], Jaime Gongora[1,*]

[1] *Faculty of Veterinary Science, RMC Gunn Building, University of Sydney, Sydney, New South Wales 2006, Australia.*

[2] *Centre for Crocodile Research, P.O. Box 329, Noonamah, Northern Territory 0837, Australia.*

[3] *Faculty of Veterinary Science, McMaster Building, University of Sydney, New South Wales 2006, Australia.*

* Corresponding author: Phone: +61-2 9036 9348. Fax: +61-2 9351 3957. E-mail: jaime.gongora@sydney.edu.au

Appendix S4. BLASTN searches of MHC class II primers used in this study against genome drafts of *Alligator mississippiensis* (v0.2.1), *Crocodylus porosus* (v0.2) and *Gangeticus gavialis* (v0.2). These genome resources are available in GenBank and can be accessed with authors' permission (St John et al. 2012). Good hits were observed between the primers and putative MHC class II sequences from those species, and this is likely to suggest an unbiased amplification of the presumed targets using the current primers. All the MHC class II sequences on the genomes were annotated and are expected to be published in another manuscript (Jaratlerdsiri et al. unpublished data).

1 Query: II *α1*-F, AACGATGAGATCTTCCATGTGG

1.1 Subject: Alligator MHC class II *α* sequence 1 (Almi-DAA)

E-value	Identities	Gaps	Strand
6e-10	22/22(100%)	0/22(0%)	Plus/Plus

```
Query  1    AACGATGAGATCTTCCATGTGG  22
            ||||||||||||||||||||||
Sbjct  166  AACGATGAGATCTTCCATGTGG  187
```

1.2 Subject: Crocodile MHC class II *α* sequence 1 (Crpo-DAA)

E-value	Identities	Gaps	Strand
6e-10	22/22(100%)	0/22(0%)	Plus/Plus

```
Query  1    AACGATGAGATCTTCCATGTGG  22
            ||||||||||||||||||||||
Sbjct  166  AACGATGAGATCTTCCATGTGG  187
```

1.3 Subject: Gavial MHC class II *α* sequence 1 (Gaga-DAA)

E-value	Identities	Gaps	Strand
4e-08	19/19(100%)	0/19(0%)	Plus/Plus

```
Query  4    GATGAGATCTTCCATGTGG  22
            |||||||||||||||||||
Sbjct  169  GATGAGATCTTCCATGTGG  187
```

2. Query: II *α1*-R, GATCTGGGACCGTGTGCG

2.1 Subject: Alligator MHC class II *α* sequence 1 (Almi-DAA)

E-value	Identities	Gaps	Strand
1e-07	18/18(100%)	0/18(0%)	Plus/Minus

```
Query  1    GATCTGGGACCGTGTGCG  18
            ||||||||||||||||||
Sbjct  336  GATCTGGGACCGTGTGCG  319
```

2.2 Subject: Crocodile MHC class II α sequence 1 (Crpo-DAA)

E-value	Identities	Gaps	Strand
1e-07	18/18(100%)	0/18(0%)	Plus/Minus

```
Query  1    GATCTGGGACCGTGTGCG  18
            ||||||||||||||||||
Sbjct  336  GATCTGGGACCGTGTGCG  319
```

2.3 Subject: Gavial MHC class II α sequence 1 (Gaga-DAA)

E-value	Identities	Gaps	Strand
1e-07	18/18(100%)	0/18(0%)	Plus/Minus

```
Query  1    GATCTGGGACCGTGTGCG  18
            ||||||||||||||||||
Sbjct  336  GATCTGGGACCGTGTGCG  319
```

3. Query: II *α2*-F, GTGTTTTCGGAGGACCCTGTG

3.1 Subject: Alligator MHC class II α sequence 1 (Almi-DAA)

E-value	Identities	Gaps	Strand
1e-04	19/21(90%)	0/21(0%)	Plus/Plus

```
Query  1    GTGTTTTCGGAGGACCCTGTG  21
            |||||||| ||| ||||||||
Sbjct  355  GTGTTTTCCGAGAACCCTGTG  375
```

3.2 Subject: Crocodile MHC class II α sequence 1 (Crpo-DAA)

E-value	Identities	Gaps	Strand
6e-07	20/21(95%)	0/21(0%)	Plus/Plus

```
Query  1    GTGTTTTCGGAGGACCCTGTG  21
            |||||||| ||||||||||||
Sbjct  355  GTGTTTTCTGAGGACCCTGTG  375
```

3.3 Subject: Gavial MHC class II α sequence 1 (Gaga-DAA)

E-value	Identities	Gaps	Strand
6e-07	20/21(95%)	0/21(0%)	Plus/Plus

```
Query  1    GTGTTTTCGGAGGACCCTGTG  21
            |||||||| ||||||||||||
Sbjct  355  GTGTTTTCTGAGGACCCTGTG  375
```

4. Query: II *α2*–R, CAGCCCCCAGTGCTCCAC

4.1 Subject: Alligator MHC class II α sequence 1 (Almi-DAA)

E-value	Identities	Gaps	Strand
1e-07	18/18(100%)	0/18(0%)	Plus/Minus

```
Query  1    CAGCCCCCAGTGCTCCAC  18
            ||||||||||||||||||
Sbjct  594  CAGCCCCCAGTGCTCCAC  577
```

4.2 Subject: Crocodile MHC class II α sequence 1 (Crpo-DAA)

E-value	Identities	Gaps	Strand
1e-07	18/18(100%)	0/18(0%)	Plus/Minus

```
Query  1    CAGCCCCCAGTGCTCCAC  18
            ||||||||||||||||||
Sbjct  594  CAGCCCCCAGTGCTCCAC  577
```

4.3 Subject: Gavial MHC class II α sequence 1 (Gaga-DAA)

E-value	Identities	Gaps	Strand
1e-07	18/18(100%)	0/18(0%)	Plus/Minus

```
Query  1    CAGCCCCCAGTGCTCCAC  18
            ||||||||||||||||||
Sbjct  594  CAGCCCCCAGTGCTCCAC  577
```

5. Query: M2-U, CTCAGTGAAGCCCAAGGTG

5.1 Subject: Crocodile MHC class II β sequence 1 (Crpo-DAB1)

E-value	Identities	Gaps	Strand
2e-06	16/16(100%)	0/16(0%)	Plus/Plus

```
Query  4    AGTGAAGCCCAAGGTG  19
            ||||||||||||||||
Sbjct  375  AGTGAAGCCCAAGGTG  390
```

5.2 Subject: Crocodile MHC class II β sequence 2 (Crpo-DAB2)

E-value	Identities	Gaps	Strand
5e-04	12/12(100%)	0/12(0%)	Plus/Plus

```
Query  8    AAGCCCAAGGTG  19
            ||||||||||||
Sbjct  378  AAGCCCAAGGTG  389
```

6. Query: M2-D, GGCTGCTGTGCTCCACCTGG

6.1 Subject: Crocodile MHC class II β sequence 1 (Crpo-DAB1)

E-value	Identities	Gaps	Strand
9e-09	20/20(100%)	0/20(0%)	Plus/Minus

```
Query  1    GGCTGCTGTGCTCCACCTGG  20
            ||||||||||||||||||||
Sbjct  631  GGCTGCTGTGCTCCACCTGG  612
```

6.2 Subject: Crocodile MHC class II β sequence 2 (Crpo-DAB2)

E-value	Identities	Gaps	Strand
9e-09	20/20(100%)	0/20(0%)	Plus/Minus

```
Query  1    GGCTGCTGTGCTCCACCTGG  20
            ||||||||||||||||||||
Sbjct  630  GGCTGCTGTGCTCCACCTGG  611
```
